# Physiologically Based Pharmacokinetic Modeling of Clobazam and Stiripentol Co-Therapy in Dravet Syndrome

**DOI:** 10.3390/jpm15110549

**Published:** 2025-11-11

**Authors:** Bassma Eltanameli, Sulafa Al Sahlawi, Rodrigo Cristofoletti

**Affiliations:** 1 Center for Pharmacometrics & Systems Pharmacology, Department of Pharmaceutics, College of Pharmacy, University of Florida, Orlando, FL 32827, USA; beltanameli@ufl.edu (B.E.); sulafa.alsahlawi@ufl.edu (S.A.S.); 2 Department of Pharmaceutics, Faculty of Pharmacy, Mansoura University, Mansoura 35516, Egypt; 3 Department of Pharmaceutical Sciences, College of Clinical Pharmacy, King Faisal University, Al-Ahsa 31982, Saudi Arabia

**Keywords:** Dravet syndrome, clobazam, stiripentol, *N*-desmethylclobazam, PBPK, pediatrics, drug–drug interaction

## Abstract

**Background**: Dravet syndrome, a severe early-onset epileptic encephalopathy, is treated with multiple antiepileptic drugs such as clobazam (CLB) and stiripentol (STP), increasing the risk of drug–drug interactions (DDIs). Given the limited pediatric pharmacokinetic data, this study developed physiologically based pharmacokinetic (PBPK) models for CLB and STP to optimize dosing and assess DDI risk across pediatric age groups. **Methods**: We developed PBPK models for CLB, its active metabolite, *N*-desmethylclobazam (N-CLB), and STP in healthy adults and pediatric patients with Dravet syndrome aged two years and older. We evaluated the inhibitory effect of STP on CLB and N-CLB metabolism, accounting for CYP2C19 phenotypes. The model was extrapolated to predict drug exposure in pediatric patients under two years of age. **Results**: PBPK models for CLB, *N-CLB*, and STP successfully recapitulated observed pharmacokinetics in healthy adults and pediatric patients older than two years. Model verification against clinical DDI data showed that co-administration of STP with CLB resulted in a clinically insignificant increase in CLB exposure (C_min_ ratio = 1.77). In contrast, N-CLB exposure increased approximately 7-fold in CYP2C19 extensive metabolizers (C_min_ ratio ≈ 7) and slightly decreased in poor metabolizers (C_min_ ratio = 0.9), consistent with the CYP2C19-dependent metabolism of N-CLB. Extrapolation to pediatric patients under two years of age predicted CLB, N-CLB, and STP exposures that were comparable to older children and remained within their reported efficacy and safety margins, suggesting no major ontogeny-related effect on exposure. **Conclusions**: The PBPK model supports the safe extrapolation of CLB and STP co-administration to pediatric Dravet syndrome patients as young as six months.

## 1. Introduction

**Figure 1:**
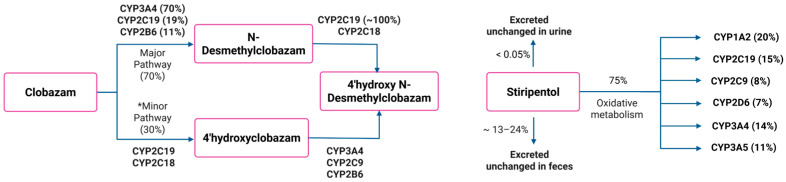
CLB and STP metabolic pathways and main metabolites. * The minor metabolic pathway also results in other metabolites, but these are not represented in the figure for simplification.

Dravet syndrome, also known as severe myoclonic epilepsy in infancy, is a genetic developmental epileptic encephalopathy that appears early in life, with an estimated prevalence of 1.2 to 6.5 per 100,000 individuals [1]. It is characterized by treatment-resistant epilepsy, with multiple seizure types that often remain poorly controlled despite polypharmacy with antiepileptic drugs. Effective seizure management in Dravet syndrome is important not only for slowing the progression of cognitive, behavioral, and developmental impairments but also for mitigating the risk of life-threatening complications, including sudden unexpected death in epilepsy and status epilepticus [1]. The management of Dravet syndrome generally requires a combination of therapies, with valproate and clobazam (CLB) being first-line treatment options [2]. The United States Food and Drug Administration (US-FDA) approved CLB in October 2011 for the treatment of seizures associated with Lennox–Gastaut syndrome in patients two years or older [3]; however, it is also commonly used off-label as an adjunct therapy in patients with Dravet syndrome [4]. CLB is mainly metabolized to the primary active metabolite *N*-desmethylclobazam (N-CLB) by cytochrome P450 (CYP450) enzymes (CYP3A4, CYP2C19, and CYP2B6), and N-CLB is further metabolized by CYP2C19 and CYP2C18 to an inactive metabolite (Figure 1) [5]. N-CLB has a longer elimination half-life (t_1/2_) compared to CLB; therefore, it takes 3 weeks to reach a steady state in healthy individuals [6]. This prolonged half-life contributes to its accumulation during chronic dosing and is believed to play a major role in seizure control during long-term therapy [7].

Since first-line treatment options in Dravet syndrome often provide partial seizure control, additional therapeutic strategies are often necessary. In 2008, the US-FDA granted orphan drug designation to stiripentol (STP) as an add-on therapy to valproate and CLB in patients aged two years and older [2]. Although the complete metabolic profile of STP has not been fully elucidated, available data indicate that oxidative metabolism accounts for approximately 75% of its total metabolism [8]. CYP phenotyping studies have identified several cytochrome P450 enzymes involved in its clearance, including CYP1A2, CYP2C19, CYP2C9, CYP2D6, and CYP3A (Figure 1). STP exhibits both direct and indirect antiepileptic activity. It enhances GABAergic transmission through a barbiturate-like mechanism, thereby amplifying inhibitory neurotransmission [9]. Additionally, STP inhibits multiple CYP450 enzymes, including CYP1A2, CYP2C9, CYP2C19, CYP2D6, and CYP3A4, in human liver microsomes at clinically relevant concentrations. This inhibition leads to elevated plasma concentrations of co-administered antiepileptic drugs and results in a synergistic antiepileptic effect [10].

With advances in genetic testing, Dravet syndrome is now often diagnosed shortly after the first seizure. Dravet syndrome typically begins between 1 and 20 months of age [11], and by 18 months, nearly 80% of patients experience status epilepticus, a life-threatening event associated with increased mortality, developmental delays, and reduced quality of life [12]. Therefore, early treatment plays a key role in improving long-term prognosis. Although the efficacy of STP has been demonstrated in two randomized, placebo-controlled trials in Dravet syndrome patients aged 3 years and older [8,13], very few therapies have been evaluated in patients under 2 years of age, despite the potential benefits of early intervention and the clear unmet medical need [14]. This is mainly due to the low frequency but high severity of seizures in this age group, which presents significant challenges for conducting randomized controlled trials. In 2022, the US-FDA expanded STP’s indication to include children as young as 6 months who are taking CLB, with the same dosing recommendations as for older children [15]. This extension was based on retrospective real-world data showing promising efficacy and acceptable safety in infants, consistent with outcomes observed in older pediatric populations [16]. However, the available data are often pooled across age groups, and infant-specific exposure data remain scarce. This is especially concerning given that developmental changes in enzyme expression and activity during infancy can significantly impact drug metabolism and overall exposure. To address these knowledge gaps, this study aims to develop and validate physiologically based pharmacokinetic (PBPK) models for CLB and STP in healthy adults and pediatric Dravet syndrome patients aged 2 years and above. These models will be used to characterize the pharmacokinetics of each drug and quantify STP-mediated increases in CLB and N-CLB exposure in CYP2C19 extensive and poor metabolizers. Once verified, the models will be extrapolated to predict STP, CLB, and N-CLB exposures and DDI risk in patients under two years of age, ultimately supporting optimized dosing strategies and enhancing the safety of CLB-STP co-therapy in this vulnerable population.

## 2. Materials and Methods

**Figure 2:**
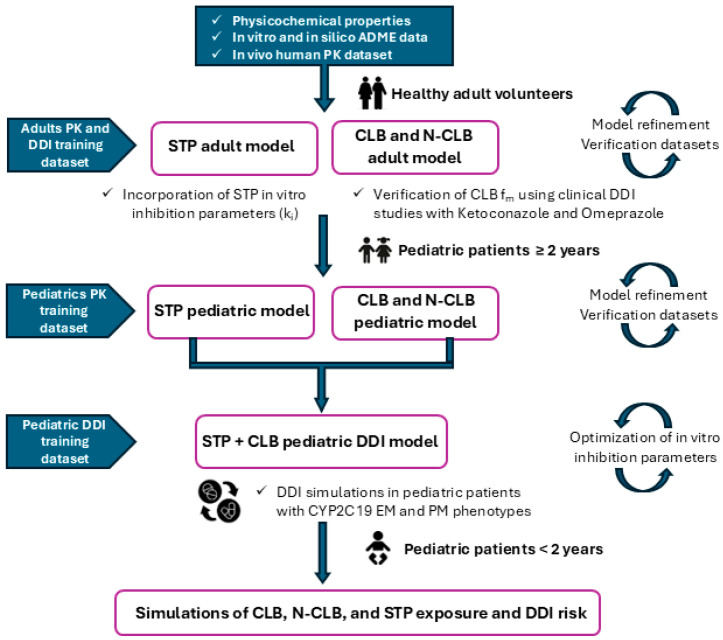
PBPK modeling workflow for CLB and STP. Abbreviations: ADME, Absorption, Distribution, Metabolism, and Excretion; EM, extensive metabolizers; f_m_, fraction metabolized; PM, poor metabolizers.

PBPK models for CLB and STP were developed using the Simcyp Simulator (Version 22, Certara UK Limited, Sheffield, UK). Compound files for CLB and STP were created by integrating physicochemical, biopharmaceutical, and pharmacokinetic parameters from the literature. When data were unavailable, parameters were either estimated by fitting to clinically observed data using Simcyp’s parameter estimation tool or predicted from molecular structures using the built-in pre-trained machine learning model in GastroPlus, ADMET Predictor (version 10.4). Since no intravenous pharmacokinetic data for CLB and STP in healthy adults were available in the literature, the model development was based on data from studies reporting the pharmacokinetics of CLB and STP following oral administration. Plasma concentration-time data reported in studies were digitized using WebPlotDigitizer (version 4.8). When observed pharmacokinetic parameters were not reported, noncompartmental analysis (NCA) was conducted using the PKNCA R package (version 0.11.0) to derive exposure metrics [17]. Figure 2 outlines the modeling workflow used to develop CLB and STP PBPK models and apply the respective models toward assessing exposure and DDI risks in pediatric patients.

### 2.1. Development and Validation of the PBPK Model for CLB and Its Active Metabolite N-CLB in Healthy Adults

The PBPK model was developed for CLB and N-CLB using a 20 mg single oral dose in fast and fed conditions [18,19] and 10 mg multiple-dose administration [19,20]. CLB blood-to-plasma ratio (B/P) was predicted by the ADMET predictor. CLB absorption was modeled using the first-order absorption model, as CLB is rapidly absorbed. The absorption rate constant (k_a_) was calculated from the reported absorption t_1/2_ to be 2.11 in the fasted condition [21] (see Supplementary Materials for calculations) and fit to 1.25 in the fed condition to capture the delay in the C_max_ [18,22]. Based on a mass balance study using [^14^C]-labeled CLB showing 81–97% recovery in urine [23] and clinical reports of ~87% oral bioavailability, the fraction absorbed (f_a_) was set to be 0.93, consistent with CLB’s extensive absorption and low first-pass extraction [23]. CLB exhibits high in vivo plasma protein binding with approximately 90% bound; thus, the unbound fraction in plasma (f_u,p_) was set to be 0.1 [24]. Due to the lack of evidence of gut wall metabolism, and consistent with the reported CLB high bioavailability, the unbound fraction in the gut (f_u,gut_) was assumed to be similar to the f_u,p_. Furthermore, a sensitivity analysis was performed to evaluate the impact of this parameter on the PBPK model outputs [25]. A full PBPK distribution model with a perfusion-limited organ model was applied to all tissues, and the Rodgers and Rowland method was used to predict the tissue to plasma partition coefficients (K_p_) and the volume of distribution at a steady state (V_ss_) for CLB. Observed CLB oral clearance (CL_po_) ranges from 1.9 to 2.3 L/h, and since CLB is extensively metabolized in the liver with only 3% excreted unchanged [3,26], the total hepatic plasma clearance (CL_h,p_) was set to be 2 L/h with renal clearance of 0.05 L/h [26]. The well-stirred liver model was used to back-calculate the hepatic intrinsic clearance (Cl_int_). Since 70% of CLB is metabolized to N-CLB [26,27], only 70% of CL_po_ (1.4 L/h) was used to characterize the Cl_int_ values for metabolizing CLB to N-CLB. An additional systemic clearance of 0.6 L/h was added to the model to account for CLB metabolism to 4-hydroxyclobazam (minor pathway). The enzyme contribution used was 70%, 19%, and 11% for CYP3A4, CYP2C19, and CYP2B6, respectively, based on in vitro chemical inhibition studies [Table S1] [5].

For N-CLB, the V_ss_ and CL_po_ were determined by noncompartmental analysis (NCA) using PKPlus^®^ (Simulation Plus, Lancaster, CA, USA) based on digitized plasma concentration-time profiles following the oral administration of 30 mg N-CLB to healthy subjects [Table S2] [28]. A K_p_ scalar of 0.8 was applied to recapitulate the observed V_ss_ [28]. The calculated CL_po_ of 1.09 L/h and CL_R_ of 0.08 L/h [28] were used to back-calculate Cl_int_, assuming that N-CLB is exclusively metabolized by CYP2C19 [5,29]. The N-CLB B/P ratio was predicted by the ADMET predictor, and f_u,gut_ was considered to be similar to f_u,p_ of 0.11 estimated in vivo [24]. The final PBPK model parameters of CLB and N-CLB are presented in Table 1. CLB and N-CLB PBPK model performance was assessed by recapitulating datasets used to build the models [19,20,22] and additional independent datasets from multiple published studies that were not used for model development [3,18,21,23,27,28,30,31,32,33,34,35,36,37]. 

To validate the enzyme contribution used in the CLB and N-CLB PBPK models in healthy adults, CLB was used as a victim drug in DDI simulations. DDI simulations were conducted using two PBPK models available in the Simcyp library, ketoconazole (a CYP3A competitive inhibitor) and omeprazole (a CYP2C19 mechanism-based inhibitor), to ensure consistency with clinical DDI study results [37]. Ketoconazole and omeprazole compound models were used without modification of default parameters. Plasma concentration-time profiles were simulated, and the AUC ratios were defined as AUC _with inhibitor_/AUC _without inhibitor_. In each DDI scenario, the simulated pharmacokinetic parameters of CLB and N-CLB, as well as the predicted AUC and C_max_ ratios, were compared to published clinical data [37].

### 2.2. Development and Validation of the PBPK Model for STP in Healthy Adults

The STP PBPK model for healthy adults was developed using data from Peinge et al. [39], in which single oral doses of 500, 1000, and 2000 mg were administered. For the multiple-dose regimen, model development was based on the study conducted by Morrison et al. [33], in which STP was administered at 750 mg twice daily for 14 days. Since STP is well-absorbed after oral administration [8], its absorption was modeled using the first-order absorption model. The f_a_ was set to 0.82, based on an in vivo mass balance study reporting that only 18% of a single 1200 mg oral dose was recovered unchanged in feces [40]. k_a_ was optimized to capture the observed C_max_ in Peinge et al. [39], and a lag time of 0.5–1.5 h was incorporated to reflect the t_max_ reported [8]. STP is highly bound to plasma proteins, with a f_u,p_ of 0.01 [41]. Due to the absence of data on the gut wall metabolism of STP, the f_u,gut_ was assumed to be equivalent to the f_u,p_, and a sensitivity analysis was conducted to evaluate the impact of this parameter on the model [25]. A full perfusion-limited PBPK model was implemented to describe the tissue distribution of STP, and the Rodgers and Rowland method was used to estimate V_ss_ and tissue K_p_ values. The predicted V_ss_ was 0.45 L/kg; however, this value did not adequately capture the distribution phase of the plasma concentration-time profile. To improve the model fit, a K_p_ scalar of 4.2 was applied, resulting in a corrected V_ss_ of 1.74 L/kg that better recapitulated the observed distribution kinetics. The K_p_ scalar was derived using the K_p_ Scalar from the Species tool in Simcyp, based on the approach described by Yun et al. [42]. This method enables the derivation of a human K_p_ scalar by comparing predicted and experimentally measured V_ss_ values in animals. Specifically, the V_ss_ of STP following intravenous administration in rhesus monkeys has been reported as 1.03 L/kg [43]. The predicted monkey V_ss_ was therefore adjusted using a K_p_ scalar of 4.2 to match the observed value. The same scalar was subsequently applied to humans, under the assumption that the K_p_ scalar in humans and animals is similar. STP undergoes extensive first-pass metabolism, with approximately 73% of the administered dose recovered in urine as metabolites, while less than 0.05% excreted unchanged in urine [40]. The oral clearance of STP markedly decreases at higher doses and following multiple-dose administration [44]. This non-linearity is primarily driven by reduced clearance, leading to a more-than-proportional increase in AUC and a prolonged half-life with ascending doses of STP [39]. Therefore, the plasma concentration-time profile following each dose level was fitted to obtain the respective dose-dependent CL_po_ [Table S3]. Given the minimal renal clearance of unchanged STP, we assumed that CL_po_ primarily reflects the total clearance. Subsequently, the obtained CL_po_ was partitioned based on the reported enzyme contributions (f_m_) to STP metabolism, using the well-stirred liver model to obtain their enzyme-specific Cl_int_ values. As per the FDA clinical pharmacology review, the key enzymes involved in STP metabolism are CYP1A2 (20%), CYP2C19 (15%), CYP2C9 (8%), CYP2D6 (7.5%), CYP3A4 (14%), and CYP3A5 (11%) [8]. We also examined the alternative possibility that the observed non-linearity was absorption-related. However, adjusting the f_a_ for each dose level while keeping clearance constant failed to adequately capture the increased exposure at higher doses, suggesting that changes in f_a_ alone cannot account for STP’s non-linear pharmacokinetics. To assess the risk of DDIs precipitated by STP, in vitro inhibition parameters for STP on various CYP450 enzymes and transporters were incorporated into the model. When multiple studies reported different k_i_ values for the same enzyme or transporter, the lowest value was selected to represent the worst-case scenario. The in vitro CYP2C19 k_i_ value of 0.139 µM for STP initially underestimated the magnitude of the clinical DDI observed between STP and N-CLB [13,45,46]. A sensitivity analysis-guided adjustment of this value (10-fold reduction to 0.0139 µM) was applied to better capture the clinically observed DDIs. This adjustment is also consistent with prior evidence showing that fluvoxamine, a potent CYP2C19 inhibitor, exhibited an in vivo inhibition potency approximately 40-fold greater than predicted from in vitro data [47]. Accordingly, the CYP2C19 k_i_ value of fluvoxamine in the Simcyp compound library is reduced approximately 9-fold to align with clinical data, consistent with our adjustment for STP. The final PBPK model parameters of STP are summarized in Table 2. To validate the STP PBPK model, its performance was evaluated using independent datasets that were not included in model development. A study involving 300, 600, and 1200 mg of single oral doses was used to assess the model’s predictive accuracy for single-dose administration [41], and three studies [10,41,44] were used to evaluate the model’s performance following multiple-dose administration across different dosing regimens.

### 2.3. Simulation Trial Design for CLB and STP PBPK Models

The trial design for each simulation was matched to the reported clinical trial design, including demographic and dosing regimen. Each simulation consisted of 10 virtual trials, but when fewer than 10 subjects were reported in a clinical trial, the number of virtual trials increased to 20 to enhance robustness. For CLB, the PBPK model was used to conduct simulations for clinical studies, including single doses ranging from 10 to 40 mg and multiple doses ranging from 5 to 40 mg in healthy volunteers (see Supplementary Tables S3 and S4). All simulations were conducted under fasting conditions unless the clinical trials explicitly indicated fed conditions. For STP, simulations were performed for doses ranging from 300 to 2000 mg, administered as single or multiple doses to healthy volunteers. According to the FDA label, STP must be taken with food due to its rapid degradation in acidic environments. Therefore, all simulations were performed under fed conditions unless the clinical trials explicitly reported administration under fasting conditions. The PBPK models were deemed acceptable when the predicted exposure metrics, including C_max_, C_min_, AUC, as well as the AUC ratio, C_max_ ratio, and C_min_ ratio, were within 2-fold of the observed summary-level clinical data. The 2-fold error margin is generally considered acceptable for most drugs when evaluating model predictability of pharmacokinetic parameters [50]. Further validations were conducted through visual inspection of the concentration-time profiles, ensuring that the simulated plasma concentrations fall within the 95% confidence intervals of the observed clinical data.

### 2.4. Development and Validation of CLB and STP PBPK Models in Pediatric Patients Above the Age of Two

The PBPK models for CLB and STP were extrapolated to pediatric populations while keeping drug-specific parameters constant. We replicated the control arms of studies that evaluated STP add-on therapy in pediatric patients with Dravet syndrome who were already stabilized on CLB. Simulations were performed to model CLB administration over a three-month period. For model development, one dataset was used for training [13], while two independent datasets were used for the external validation of the pediatric CLB PBPK model [45,46]. In addition, CLB and N-CLB exposures in CYP2C19 PMs were assessed by adjusting the CYP2C19 phenotype frequency in the pediatric population tab within Simcyp. For STP, model development in pediatric patients was performed across three pediatric age groups, as reported by May et al. [51], and model validation was conducted using the clinical study by Chiron et al. [13].

### 2.5. Metabolism-Mediated DDI Risk Assessment of CLB and STP Co-Therapy in Pediatric Dravet Syndrome Patients

To evaluate the inhibitory effect of STP on CLB and N-CLB, we simulated three studies in which STP was added to the treatment regimen of Dravet syndrome patients who were already receiving CLB [13,45,46]. In the STICLO trial, patients 3 years and older received CLB up to a maximum of 0.5 mg/kg/day for one month, followed by the addition of STP at a dose of 50 mg/kg/day administered in two divided doses for an additional two months [13]. A clinical trial conducted in Japanese patients evaluated the addition of STP at 50 mg/kg/day in patients aged 1–24 years who were stabilized on CLB up to the maximum tolerated dose of 0.5 mg/kg/day [45]. Notably, three of these patients were CYP2C19 PMs [45]. In a retrospective analysis, trough blood levels were collected from Japanese patients receiving combined therapy with a mean CLB dose of 0.23 mg/kg/day and a mean STP dose of 35 mg/kg/day [46]. In this study, 10 patients were identified as CYP2C19 PMs [46]. The reported mean age was 9.6 ± 8.8 years (range: 1–40 years); however, simulations were performed for ages 1–25 years, as 25 is the maximum age considered for the pediatric population in Simcyp. Since CLB dosing in pediatrics is weight-based and individualized according to efficacy and tolerability, C_min_ values were dose-normalized. We then compared the simulated dose-normalized trough concentrations of CLB and N-CLB in the presence of STP with observed data in both CYP2C19 EMs and PMs.

### 2.6. Extrapolation of the Validated PBPK Models of CLB and STP to Pediatric Patients Below the Age of Two

To predict systemic exposure of CLB, N-CLB, and STP in pediatric patients under two years of age, virtual clinical trials were developed using the dosing regimen described in the STICLO trial [13]. Simulations were performed in five pediatric age groups (2 years, 1.5 years, 1 year, 9 months, and 6 months), with ten trials per group, each including ten subjects with a 1:1 female-to-male ratio. Plasma concentration-time profiles and average steady-state concentrations of STP, CLB, and N-CLB were compared to the reference group of pediatric patients aged <6 years [51]. Because the concentration–dose relationship for STP is non-linear, we used a CL value of 14 L/h, consistent with that applied for the <6-year age group in May et al. [Table S3]. Age-related variability in drug disposition and interaction risk was accounted for using the default Simcyp pediatric population library, which implements ontogeny functions that describe the dynamic changes in enzyme expression and activity from birth to adulthood. These ontogeny functions are derived from in vitro and clinical data for CYP1A2 and CYP3A4 [52], CYP2B6 [53,54,55], CYP2C19 and CYP2C9 [56,57,58], and CYP2D6 [53,59,60].

### 2.7. Global Sensitivity Analysis of CLB and STP PBPK Models

We conducted a global sensitivity analysis (GSA) in Simcyp to assess the influence of optimized and fitted parameters on the developed CLB and STP PBPK models. The primary pharmacokinetic metrics of interest were C_max_ and AUC. To reduce computational complexity, a two-step approach was employed as recommended by McNally et al. [61]. This consisted of preliminary qualitative screening using the Morris method to rank the parameters based on their impact on model outputs. This method begins by sampling values for all input parameters within their predefined, physiologically relevant ranges and calculating the corresponding model output. In the subsequent step, the value of a single parameter is changed, and the resulting change in the model output is compared to the previous run. The μ* (absolute mean) is used to measure the overall effect of a parameter on the model output, with higher μ* values indicating parameters with a more substantial influence on model outputs. Subsequently, the parameters with the highest impact were selected for the quantitative GSA using the Sobol method. This method is a more detailed, variance-based technique that quantifies the contribution of each parameter and its interactions to the total variance in the model output. It generates two types of sensitivity measures: the first-order (S1) and total effect (ST) indices. The S1 sensitivity index evaluates the individual impact of a parameter without considering interactions with others, while the ST index assesses the impact of each parameter, including all potential interactions. Parameters with a sensitivity index greater than 0.1 (10%) were considered key parameters with a significant impact on the model outputs [62]. For the CLB PBPK model, the selected parameters for GSA were pKa, B/P, logP, f_u,p_, f_a_, k_a_, and f_u,gut_, whereas, for the STP model, the parameters with the highest uncertainty were f_a_, f_u,gut_, k_a_, and the K_p_ scalar.

## 3. Results

### 3.1. Development and Validation of the PBPK Model for CLB and Its Active Metabolite N-CLB in Healthy Adults

**Figure 3:**
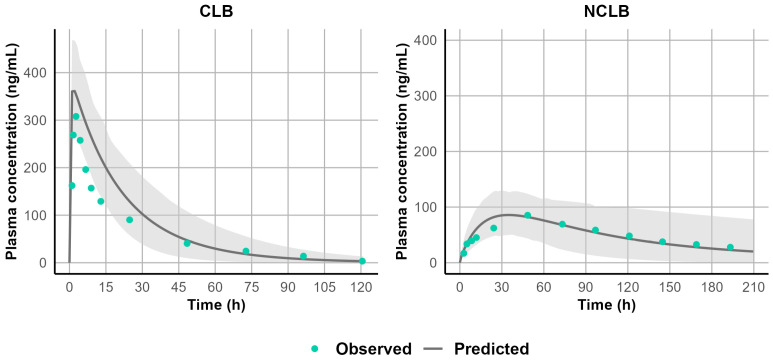
Simulated versus observed plasma concentration-time profile of CLB and N-CLB following a single 20 mg dose [19]. The solid black line represents the simulated mean concentration profile, and the gray-shaded area represents the simulated 5th to 95th percentile. Green circles represent observed concentrations.

**Figure 4:**
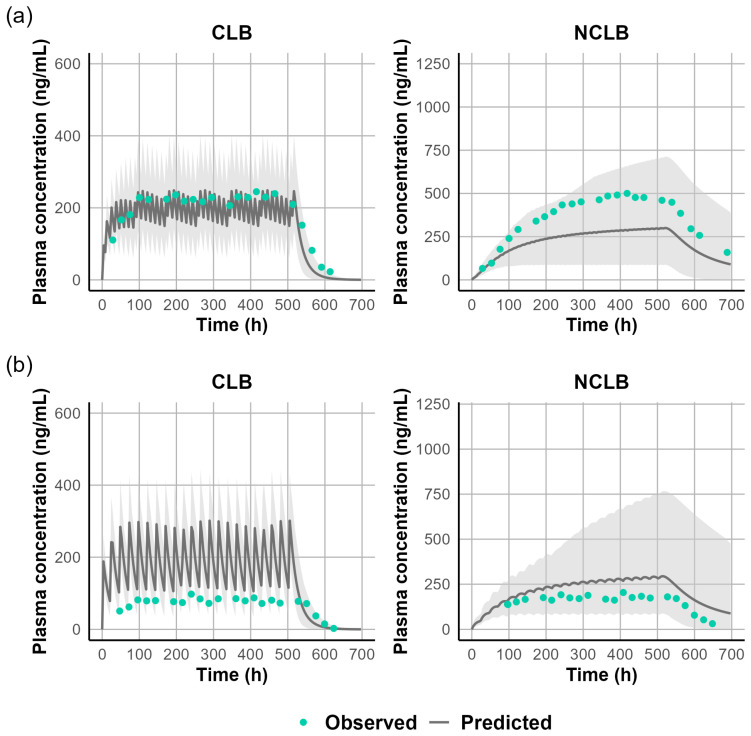
Simulated versus observed steady-state plasma concentration-time profiles (C_ssmin_) of CLB and N-CLB following oral administrations of (**a**) CLB 5 mg twice daily [34] or (**b**) CLB 10 mg once daily [20]. The solid black line represents the simulated mean concentration profile, and the gray-shaded area represents the simulated 5th to 95th percentile. Green circles represent observed concentrations.

**Figure 5:**
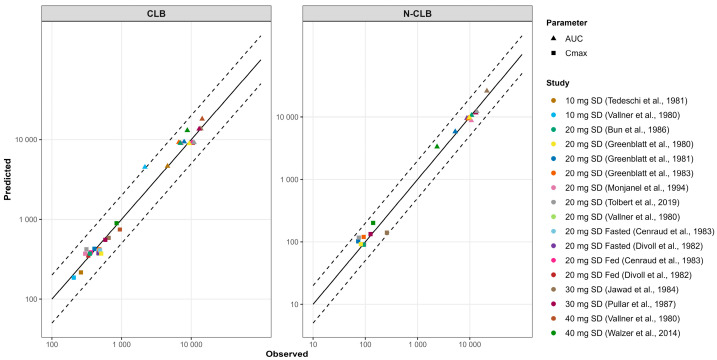
Goodness-of-fit plots of predicted versus observed pharmacokinetic metrics (AUC and C_max_) for CLB and N-CLB across various clinical studies [3,18,19,20,21,22,28,30,31,32,35,36,37]. The solid and dashed lines represent the identity line and 2-fold deviation, respectively. Abbreviations: SD, single dose.

Because the in vitro–in vivo extrapolation (IVIVE) of Cl_int_ overpredicted CLB clearance and underpredicted N-CLB clearance, the well-stirred liver model was used to estimate CLB and N-CLB Cl_int_ using enzyme contribution from in vitro chemical inhibition studies for CLB [5]. The Cl_int_ values for CYP 3A4, 2C19, and 2B6 were 0.019, 0.173, and 0.022 µL/min/pmol P450. Using these Cl_int_ values resulted in capturing the CLB in vivo CL. The well-stirred liver model was also used to estimate N-CLB Cl_int,_ which was estimated to be 0.636 µL/min/pmol. Figure 3 and Figure 4 illustrate the model simulations with observed clinical data of the single-dose [19] and multiple-dose administrations [20,34] used for model development, demonstrating the internal validity of the CLB PBPK model. Further assessment of the CLB and N-CLB PBPK models’ performances is shown in Table S4 and Table S5, respectively. Overall, the ratio between simulated and observed values for AUC and C_max_ was within a 2-fold range except for Rupp et al., who reported significantly higher N-CLB steady-state concentrations after 10 mg twice daily (BID). The authors observed levels of N-CLB that were approximately eight times higher than the steady-state concentrations of CLB [23], whereas other clinical studies have typically reported N-CLB/CLB ratios of 3–5 at a steady state. Figure 5 presents the goodness-of-fit plots comparing predicted and observed AUC and C_max_ values for CLB and N-CLB, indicating that the model adequately captured the clinical data used to validate the model externally.

When simulating the drug interaction risk in healthy adults using CLB as a substrate and ketoconazole as a CYP3A4 inhibitor, the model successfully predicted the CLB plasma profiles and the extent of DDI with CLB. On the other hand, when simulating the interaction between N-CLB and ketoconazole, the model predicted the N-CLB plasma profiles, but there was a slight underprediction of the extent of the DDI with an AUCR value of 0.8, in contrast to the observed value of 1.15. Both CLB and N-CLB PBPK models effectively predicted the plasma concentrations and extent of DDIs with the CYP2C9 inhibitor, omeprazole, where AUCR was predicted to be 1.2, consistent with the observed value of 1.36. In both cases, the DDI simulations did not predict any significant interaction with either ketoconazole or omeprazole, which is consistent with the findings of the published clinical DDI studies (see Supplementary Tables S3 and S4).

### 3.2. Development and Validation of the PBPK Model for STP in Healthy Adults

**Figure 6:**
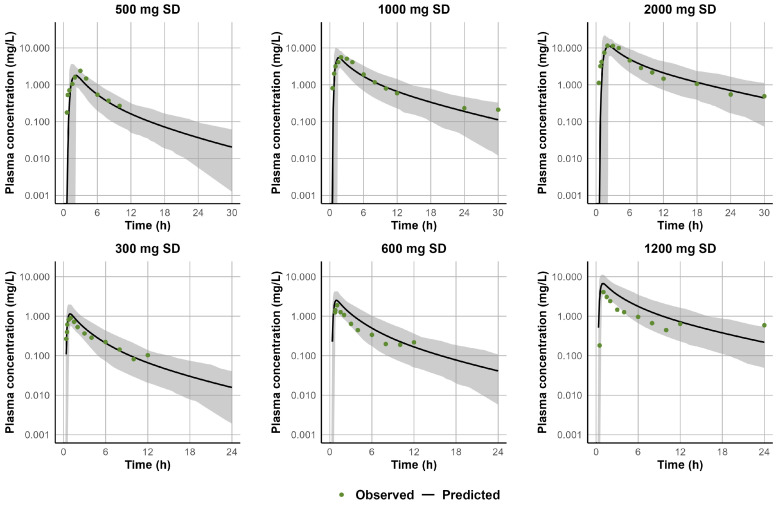
Simulated versus observed plasma concentration-time profiles of STP following single-dose administration. The upper row represents doses of 500, 1000, and 2000 mg used to train the model [39], while the lower row corresponds to 300, 600, and 1200 mg used to externally validate the model [41]. The solid black line represents the simulated mean concentration profile, and the gray-shaded area represents the simulated 5th to 95th percentile. Green circles represent observed data from the respective studies.

**Figure 7:**
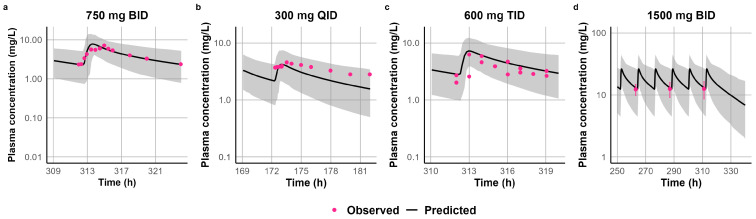
Simulated versus observed plasma concentration-time profiles of STP following multiple-dose administration. (**a**) Treatment of 750 mg twice daily used to train the model [33]; model external validation using (**b**) 300 mg four times daily [41]; (**c**) two patients receiving 600 mg three times daily [44]; and (**d**) 1500 mg twice daily [10]. The solid black line represents the simulated mean concentration profile, and the gray-shaded area represents the simulated 5th to 95th percentile. Pink circles represent observed data from the respective studies.

The predicted CL_po_ for STP ranged from 8 to 70 L/h, showing a clear trend of reduced clearance at higher doses and following chronic administration. Table S3 summarizes the fitted CL_po_ values that were subsequently used in the well-stirred liver model to back-calculate the enzyme-specific CL_int_ incorporated into the PBPK model. Following single-dose administration, the 500 mg dataset used for model development was validated against the 600 mg dataset with a CL_po_ of 60 L/h. However, this value overpredicted exposure at 300 mg, requiring an increase in CL_po_ to 70 L/h. The 1000 mg dataset used for model development was successfully validated using the 1200 mg dataset with a CL_po_ of 35 L/h. For the 2000 mg dataset, a CL_po_ of 25 L/h was applied; however, this could not be independently verified due to the lack of a dataset at a comparable dose. For multiple-dose administration, the model was developed and validated with a CL_po_ of 17 L/h, which adequately captured exposures across the 1200–1800 mg/day dose range when administered as divided doses. At higher doses of 1500 mg administered twice daily, clearance was further reduced to 8 L/h; however, this adjustment could not be externally validated due to limited pharmacokinetic data at this dose level. Overall, the model recapitulated the observed clinical data, demonstrating predictive accuracy across a range of single and multiple doses. Figure 6 and Figure 7 illustrate the alignment between simulated and observed plasma concentration-time profiles following single- and multiple-dose administration, respectively. In addition, the simulated-to-observed ratios for AUC and C_max_ consistently fell within the acceptable range of 0.5–2.0 (Figure S1). A detailed comparison of simulated and observed pharmacokinetic parameters (e.g., C_max_, AUC) across the clinical studies used for model development and verification is provided in Table S6.

### 3.3. Development and Validation of CLB and STP PBPK Models in Pediatric Patients Above the Age of Two

**Figure 8:**
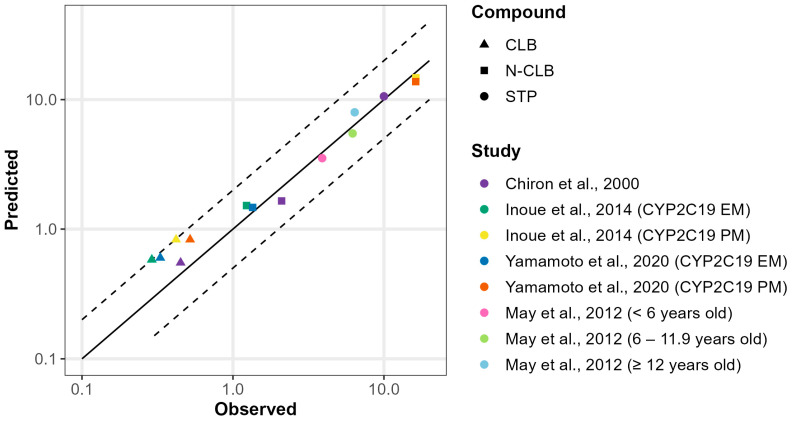
Goodness-of-fit plot of predicted versus observed minimum concentration (C_min_) for CLB, N-CLB, and STP across various clinical studies in pediatrics [13,45,46,51]. The solid and dashed lines represent the identity line and 2-fold deviation, respectively.

In the pediatric population, our PBPK models demonstrated good predictive performance across various age groups receiving weight-based dosing regimens. For STP, CL_po_ decreased in older children, consistent with the higher absolute doses administered under weight-based dosing. To capture STP’s non-linearity, CL_po_ values were adjusted from 14 L/h in patients < 6 years to 8 L/h in those 12 years and above [Table S3]. For the study by Chiron et al., which included a wide age range with a mean of 9.4 [3–16.7] years, a CL_po_ of 8 L/h was applied [Table S3]. Our models recapitulated CLB, N-CLB, and STP trough concentrations (C_min_) within 2-fold of the observed values in Dravet syndrome pediatric patients (Figure 8; Table S7). In CYP2C19 PMs, CLB exposure showed a modest increase, whereas N-CLB, which is exclusively metabolized by CYP2C19, exhibited a substantial 10-fold increase, in agreement with clinically observed data [45,46].

### 3.4. Metabolism-Mediated DDI Risk Assessment of CLB and STP Co-Therapy in Pediatric Dravet Syndrome Patient

The PBPK model successfully recapitulated CLB and N-CLB trough concentrations in pediatric patients with Dravet syndrome aged one year and older when STP was co-administered. Simulated-to-observed ratios for C_min_ in the presence of STP, as well as C_min_ ratios, were within the predefined acceptance range of 0.5–2.0. No clinically relevant interaction between CLB and STP was observed in either CYP2C19 EMs or PMs, with C_min_ of CLB in the presence and absence of STP remaining below 2. In contrast, STP markedly increased N-CLB trough concentrations in CYP2C19 EM, with a 7–8-fold elevation due to CYP2C19 inhibition. This finding is consistent with clinical studies showing a 4–7-fold increase in N-CLB exposure in the presence of STP [13,45,46]. In CYP2C19 PMs, however, this effect was absent; instead, N-CLB levels decreased slightly (0.9-fold), in line with the clinically reported 0.6-fold reduction in N-CLB exposure when STP was co-administered [13,45,46]. Table 3 and Figure S3 summarize the results of the DDI simulations involving CLB and STP in Dravet syndrome pediatric populations.

### 3.5. Extrapolation of the Validated PBPK Models of CLB and STP to Pediatric Patients Below the Age of Two

**Figure 9:**
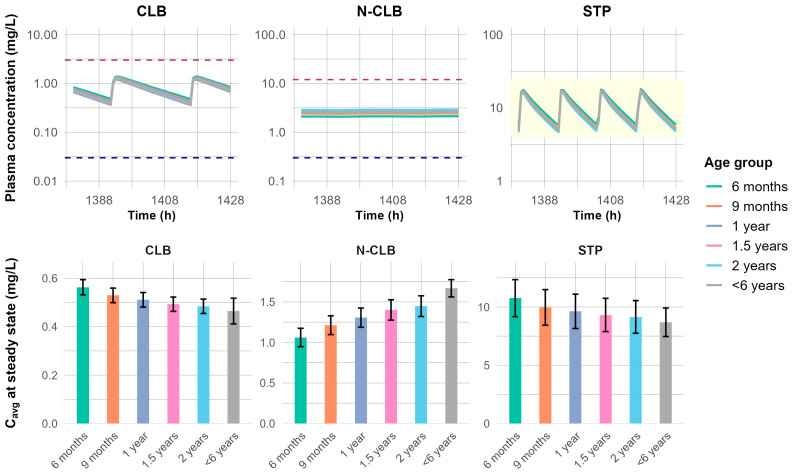
Simulated steady-state concentrations of CLB, N-CLB, and STP across various pediatric age groups. The top panel shows the concentration-time profile at a steady state. The yellow shaded area represents STP clinically effective plasma concentrations [45,64], the red dashed line represents the toxic levels for CLB and N-CLB [6], and the blue dashed line represents the minimal therapeutic levels for CLB and N-CLB [6,63]. The bottom panels show the average concentration (C_avg_) at steady state, with bars representing mean concentrations and error bars indicating the standard deviation (SD).

Clinical trial simulations were conducted to predict systemic exposure of CLB, N-CLB, and STP when STP was added to ongoing CLB therapy in pediatric patients younger than two years of age. Simulated plasma concentration-time profiles of CLB, N-CLB, and STP at a steady state were comparable across all evaluated pediatric age groups (6 months, 9 months, 1 year, 1.5 years, 2 years, and <6 years; Figure 9), suggesting modest ontogeny-related differences under the proposed dosing regimen. Average CLB concentrations showed slight variation with age, ranging from 0.48 to 0.56 mg/L in children 6 months to 2 years, comparable to the reference group of <6 years (0.47 mg/L). In contrast, N-CLB exposure increased progressively with age, from 1.06 mg/L at 6 months to 1.45 mg/L at 2 years, with all values being lower than the reference group (1.67 mg/L). STP exposure demonstrated a similar trend to CLB, decreasing with age from 10.76 mg/L at 6 months to 9.15 mg/L at 2 years, comparable to the reference group level of 8.69 mg/L (Figure 9). Overall, the average steady-state concentration remained consistently within the respective therapeutic range across all simulated age groups, demonstrating predicted exposures consistent with clinically reported efficacy and safety margins [6,45,63,64]. Therefore, these findings support the extrapolation of current CLB and STP dosing recommendations to pediatric Dravet syndrome patients aged ≥6 months.

### 3.6. Global Sensitivity Analysis of CLB and STP PBPK Models

**Figure 10:**
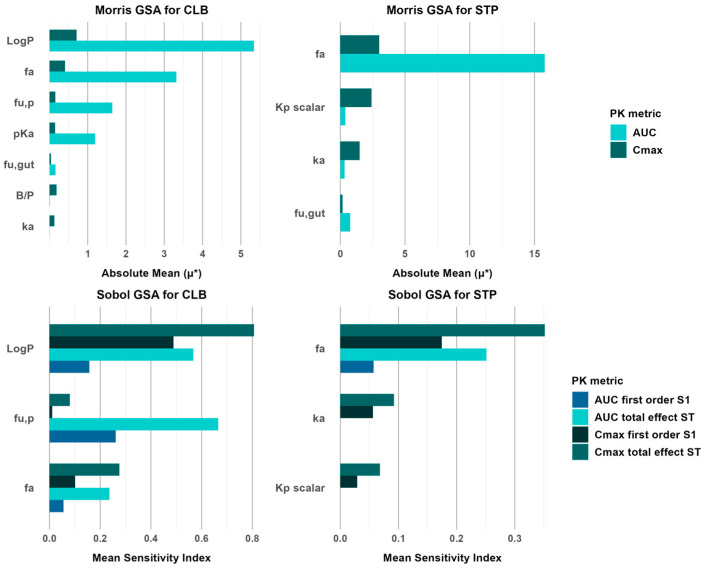
Global Sensitivity Analysis results of CLB and STP PBPK models. The top row (Morris method) shows parameters ranked by their absolute mean (μ*) values, where a higher μ* indicates a more significant influence on model outputs (AUC, C_max_). The bottom row (Sobol method) shows first-order and total-effect sensitivity indices for AUC and C_max_, with higher indices indicating greater parameter importance for the model.

The Morris GSA results for CLB indicate that logP has the highest sensitivity effect on AUC, followed by f_a_, both of which significantly affect drug exposure. Additionally, f_u,p,_ and pKa show a moderate influence on AUC. On the other hand, C_max_ demonstrates generally lower sensitivity across all tested parameters, with logP and f_a_ having only minor effects. For the STP model, the analysis indicates that f_a_ is the most influential parameter for both AUC and C_max_. Meanwhile, for C_max_, the K_p_ scalar and k_a_ show a moderate influence, suggesting their role in peak concentration variability. Figure 10 presents the qualitative ranking of parameter influence on CLB and STP C_max_ and AUC relative to their absolute mean values (μ*).

The Sobol GSA results for the CLB model support the key parameters identified in the Morris GSA, with logP, f_a_, and f_u,p_ exhibiting the highest influence on the pharmacokinetic metrics. For the STP model, the Sobol analysis confirms that f_a_ remains the most influential parameter for both AUC and C_max_, with a sensitivity index exceeding the 0.1 (10%) threshold based on the total-effect sensitivity index (St). In contrast, k_a_ and K_p_ scalar show no significant impact on AUC and C_max_. Figure 10 presents the Sobol sensitivity analysis results, showing the first-order sensitivity index (S1) and total-effect sensitivity index (St) for key parameters in relation to AUC and C_max_.

## 4. Discussion

In this study, we developed and validated CLB, N-CLB, and STP PBPK models that recapitulated systemic exposure after single and multiple doses across adult and pediatric populations (Figure 3, Figure 4, Figure 5, Figure 6, Figure 7 and Figure 8) [18,19,20,21,22,26,28,30,34,36,37,39,41,65]. The only exception was the study by Rupp et al. [23], which reported significantly higher N-CLB concentrations than other studies in the literature. Across other clinical studies, steady-state N-CLB concentrations were approximately 3–5-fold higher than CLB [6,38], whereas Rupp et al. reported nearly 8-fold higher N-CLB levels in healthy subjects during multiple dosing. One possible explanation could be interindividual variability in CYP2C19 activity, which is now known to markedly influence N-CLB exposure [66]. Several studies have shown that CYP2C19 poor metabolizers exhibit 3–4-fold higher N-CLB concentrations than extensive metabolizers [3,23,38,67]. However, Rupp et al. was one of the earliest human studies with CLB, and no genotyping or phenotyping was performed, so the reason for this discrepancy remains uncertain. IVIVE underestimated CLB hepatic clearance despite ISEF (Inter-System Extrapolation Factor) correction in Simcyp; therefore, we used a middle-out back-calculation of enzyme-specific CL_int_. The resulting CLB and N-CLB PBPK models reproduce the calibration studies [19,20,22] and recapitulate additional studies [18,21,27,28,30,31,32,33,34,35,36,37] without further adjustment. These findings align with prior CLB PBPK work [68], which also reported failure to recover CL_po_ from in vitro parameters and required empirical scaling. CLB does not inhibit any major CYP450 or UGT enzyme in vitro, while N-CLB weakly inhibits CYP2C9, UGT1A4, UGT1A6, and UGT2B4 [38]. The reported inhibition constants (IC50 values > 25–62.5 µM) are substantially higher than the clinically observed plasma concentrations of N-CLB [38]. At therapeutic doses (20–40 mg/day), steady-state C_avg_ of N-CLB are typically ~3–9 µM (~850–2500 ng/mL). Therefore, these interactions were not incorporated into the PBPK model, as they are not expected to be clinically relevant, and there is no evidence for in vivo inhibition in the literature. Although CLB and N-CLB induced CYP3A4 in vitro, clinical interaction studies show only modest effects on exposure when this pathway is inhibited. Co-administration of ketoconazole 400 mg (CYP3A4 inhibitor) increased CLB AUC by ~54% with no change in C_max_ [37]. This change is not considered clinically significant and does not suggest substantial induction in vivo. CLB and N-CLB have also been reported as P-gp substrates in vitro [38]. CLB pharmacokinetics are dose-proportional and linear across the therapeutic range [38], providing no evidence that P-gp plays a significant role in its absorption. Furthermore, CLB is well-absorbed, even in the presence of food [18,22], and can penetrate the blood–brain barrier [24,26]; therefore, the in vivo influence of P-gp exposure is probably negligible [3]. Consistent with this, co-administration with ketoconazole, a known P-gp inhibitor, did not result in clinically significant changes in CLB exposure, supporting the limited contribution of P-gp to its systemic disposition [37]. Accordingly, we did not incorporate P-gp into the current PBPK model. The CLB PBPK model was further evaluated in healthy adults by using it as a victim for inhibition by ketoconazole and omeprazole. The model successfully recapitulated clinical DDI studies, confirming the fraction metabolized (f_m_) of each enzyme in our model. It should be noted that when simulating the interaction between CLB as a victim and ketoconazole as a CYP3A4 inhibitor, there was a slight underprediction of the extent of the DDI with N-CLB. This is because ketoconazole inhibited the CYP3A4 metabolic pathway, which resulted in increased CLB concentrations and decreased N-CLB concentrations. On the contrary, Walzer et al.’s study reported increased N-CLB concentrations when CLB is administered with ketoconazole [37]. The sensitivity analyses using the Morris and Sobol methods identified f_u,p_ as one of the most significant parameters affecting CLB exposure in the PBPK model. Both CLB and N-CLB are highly protein-bound, mainly for plasma albumin [24]. The in vitro plasma protein binding of CLB and N-CLB was reported to be 80–90% and 70%, respectively, and is concentration-independent [38]. However, a recent clinical study assessed the protein binding of CLB and N-CLB in patient serum and reported 90% and 89% binding for CLB and N-CLB, respectively [24]. Because CLB and N-CLB are predominantly albumin-bound, serum–plasma differences are expected to be small; therefore, these values were implemented in the PBPK model for physiological relevance.

Our PBPK model successfully captured STP’s dose-dependent Michaelis–Menten kinetics by reducing CL_po_ at higher doses. Since our primary objective was to evaluate the risk of DDIs, it was necessary to incorporate enzyme kinetics into the model. However, due to the lack of in vitro data characterizing STP metabolism, a middle-out approach was adopted as a fit-for-purpose strategy. The non-linear pharmacokinetic behavior of STP was noted in the STP FDA label, which reports a significant increase in the elimination half-life with increased doses. Similarly, studies in healthy volunteers [41,44] and in patients with epilepsy [65] demonstrated a consistent reduction in apparent clearance at escalating doses. Peigné et al. further confirmed dose-dependent non-linearity by showing a more-than-proportional increase in AUC and a prolonged half-life following doses of 500, 1000, and 2000 mg, even though dose-normalized C_max_ values did not significantly vary between doses [39]. On the other hand, a population pharmacokinetic analysis found that STP pharmacokinetics in children with Dravet syndrome were best characterized by a one-compartment model with zero-order absorption and first-order elimination [69]. These findings indicate the possibility of saturable absorption at higher doses, likely due to solubility limitations or the involvement of a saturable transporter. However, in vitro studies suggest that STP is not a substrate of major transporters, including P-gp, BCRP, OATP1B1, OATP1B3, OAT1, OAT3, or OCT2 [8]. Notably, our sensitivity analyses using the Sobol and Morris methods identified f_a_ as the most influential parameter affecting STP exposure in the PBPK model. In addition, our model addressed the effect of chronic dosing on STP clearance by reducing the CL_po_ after multiple doses to reflect the observed increase in exposure at a steady state. Levy et al. reported that, by day 8, STP’s AUC increased about 7.8-fold compared with day 1, alongside a decrease in clearance from 2.14 to 0.92 L/h/kg and an increase in the fraction of STP excreted unchanged in urine from 0.04% to 0.11% [41]. This reduction in clearance following repeated dosing can be attributed to auto-inhibition. STP’s methylenedioxyphenyl group is converted to a reactive carbene intermediate that forms a metabolic intermediate complex (MIC) with CYP enzymes, leading to time-dependent inhibition [70]. Masubuchi and colleagues confirmed STP time-dependent inhibition (TDI) of CYP1A2 via MIC formation, although they did not report the TDI parameters needed for parameterizing our PBPK model [71].

STP is a potent inhibitor of several CYP enzymes both in vitro and in vivo. When co-administered with other antiepileptic drugs, such as phenytoin, phenobarbital, and carbamazepine, STP can elevate their plasma concentrations through CYP inhibition [65]. However, these antiepileptic drugs are generally avoided in Dravet syndrome because they may worsen seizures. A more clinically relevant interaction that warrants investigation involves STP co-administration with CLB in Dravet syndrome. Chiron et al. reported approximately 1.9-fold and 5.5-fold increases in CLB and N-CLB levels, respectively, when STP was added [13]. Our DDI model successfully captured these increased CLB and N-CLB plasma concentrations in the presence of STP. The involvement of multiple metabolic pathways in CLB metabolism can explain the relatively modest interaction between CLB and STP. Meanwhile, we predicted a substantial 7-fold increase in the concentration-to-dose ratio for N-CLB upon STP co-administration, likely contributing to the enhanced seizure control observed clinically. Moreover, N-CLB exposure differs significantly across CYP2C19 phenotypes. Our simulations were consistent with clinical data, showing markedly higher N-CLB levels in PMs compared with EMs [45,46]. Interestingly, the addition of STP to CLB therapy in CYP2C19 PMs did not increase N-CLB exposure but instead reduced it, likely due to CYP2C19 dysfunction limiting further interaction and STP’s inhibition of CYP3A4 decreasing the conversion of CLB to N-CLB.

Optimizing early treatment strategies for Dravet syndrome is critical for improving long-term neurological outcomes. Chiron et al. reported their 30-year clinical experience demonstrating that initiating STP, alongside valproate and CLB as early as 4 to 24 months of age, was safe, well-tolerated, and led to significant reductions in convulsive seizures, status epilepticus episodes, and hospitalization frequency, ultimately enhancing patients’ quality of life [16]. Our PBPK modeling predictions support these clinical observations by providing quantitative evidence that systemic exposures of CLB, N-CLB, and STP in patients under two years of age are comparable to those in older children. The therapeutic reference range for STP has not been clearly established. Nonetheless, STP is generally well-tolerated, and most adverse effects typically result from DDIs and resolve after adjustment of concomitant medications. Clinically effective plasma concentrations have been reported between 4 and 25 mg/L [45,64], while no specific plasma levels associated with toxicity have been identified. On the other hand, CLB shows poor correlation between plasma concentrations and therapeutic efficacy, and no validated therapeutic range has been established [72]. The best approximation, as proposed by Patsalos et al., suggests that in patients receiving therapeutic doses of CLB, serum concentrations typically range from 0.03 to 0.3 mg/L for CLB and 0.3 to 3 mg/L for N-CLB [63]. Given CLB’s wide therapeutic window, only markedly elevated levels are associated with toxicity; concentrations above 3 mg/L for CLB and/or 12 mg/L for N-CLB are considered indicative of CLB intoxication in the presence of clinical symptoms [6]. Our simulations predicted that STP exposures remained within the effective therapeutic concentration, whereas CLB and N-CLB exposures were slightly above the proposed target concentrations but remained well below toxic thresholds.

In pediatrics, growth and maturation processes are non-linear, and simple allometric scaling often fails to reliably predict clearance, particularly in children younger than two years [73]. PBPK modeling provides a more mechanistic approach by accounting for the ontogeny of drug-metabolizing enzymes and other age-dependent physiological changes. Our findings are consistent with developmental enzyme ontogeny, whereby higher CLB and STP exposures seen in younger infants likely reflect reduced clearance due to lower expression and activity of CYP enzymes, whereas the age-dependent increase in N-CLB exposure suggests enhanced CYP2C19 activity with maturation, leading to greater metabolic conversion from CLB. N-CLB showed the most significant variability across age groups, likely due to its exclusive metabolism via CYP2C19, whose activity is approximately 30% of adult levels at birth and gradually matures to adult capacity by around 2 years of age [56]. Similar age-dependent clearance patterns have been observed for other CYP2C19 substrates, such as omeprazole and lansoprazole, where children older than one year generally display adult-like pharmacokinetics [74,75]. To verify and strengthen confidence in our predictions, we reviewed reports evaluating the performance of the Simcyp pediatric population models across compounds primarily metabolized by CYP enzymes, using the default Simcyp ontogeny profiles. Johnson et al. demonstrated that predicted clearance for nine CYP-metabolized drugs was within 2-fold of observed values in 70% of neonates, 100% of infants, and 89% of children [76]. Moreover, Zhou et al. developed PBPK models that reasonably predicted drug exposure in children aged one month and older, with 58 of 67 predictions falling within 2-fold of observed values [77]. Lastly, a review by Small et al. extended the evaluation beyond traditional probe substrates by assessing compounds with properties more representative of drugs in development, including complex disposition and diverse elimination pathways [78]. Across these compounds, 94% of pediatric pharmacokinetic parameter predictions fell within 2-fold of observed values.

We acknowledge the existence of previously published PBPK models for CLB and STP [68]; however, these were developed in MATLAB 7.14.0.739 (R2012a), which requires specialized programming expertise and lacks the built-in population and drug libraries. Unlike earlier models, in which variability was primarily driven by body weight or body surface area, our models account for interindividual variability in both drug- and system-related parameters, including the drug-metabolizing enzymes. In addition, our models incorporate physiological ontogeny, enabling the prediction of CLB, N-CLB, and STP exposure in pediatric patients as young as six months of age. The integration of enzyme kinetics in our models allowed the assessment of DDI risks and the impact of CYP2C19 genetic polymorphisms. Furthermore, the successful development and validation of these PBPK models provides a valuable tool for understanding the efficacy and safety of combination therapy in Dravet syndrome. Since many patients receive concurrent treatment with other antiepileptic drugs, incorporating enzyme contributions into our PBPK framework facilitates assessing interactions with potent CYP450 modulators. For instance, cannabidiol (CBD), an FDA-approved therapy for Dravet syndrome known for its significant inhibitory effects on several drug-metabolizing enzymes [79], represents a key candidate for future investigations. Our future work will explore the roles of CLB and STP as victim drugs when co-administered with CBD, thereby expanding the clinical utility and applicability of these PBPK models.

Our PBPK models for CLB and STP have several limitations. First, the absence of intravenous data prevents a thorough characterization of the distribution and elimination profiles of CLB and STP. Second, the N-CLB model does not incorporate time-dependent reduction in N-CLB clearance following multiple dosing [19], which may explain the observed underprediction of N-CLB exposure in multiple-dose simulations. Additionally, because STP was approved as an orphan drug, significant gaps remain in the available pharmacokinetic literature. In particular, the lack of comprehensive in vitro metabolism data for STP necessitated the fitting of different CL_po_ values for each dose level to describe the non-linearity rather than using mechanistic parameters such as V_max_ and K_m_ for the contributing metabolic enzymes. The CL_po_ values used in our model were validated against a wide range of STP doses (600–1200 mg single dose; 1200–1800 mg/day divided doses). However, due to limited clinical pharmacokinetic data, external validation could not be performed for the 300 mg, 2000 mg, and 1500 mg BID regimens. Consequently, not all dose levels were externally validated, reflecting the constraints of the available datasets. Another challenge was the limited availability of concentration-time data in pediatric populations. Retrospective pharmacokinetic studies often report only single-point steady-state concentrations (typically C_min_), which restricts the ability to rigorously validate the PBPK models against full concentration-time profiles for CLB and STP in pediatrics.

## 5. Conclusions

In conclusion, our PBPK models for CLB, its active metabolite N-CLB, and STP successfully recapitulated observed clinical trial data across single and multiple doses in both healthy adults and pediatric populations. These models also captured the DDI between CLB and STP in pediatric Dravet syndrome patients with different CYP2C19 phenotypes. Our modeling results indicate that standard dosing regimens achieve therapeutically appropriate exposures in Dravet syndrome patients as young as 6 months, supporting the safe and effective use of CLB and STP co-therapy in this population. Thus, early intervention, supported by appropriate pharmacokinetic exposure, may provide a valuable opportunity to improve clinical outcomes in these vulnerable patients.

## Figures and Tables

**Table 1:** Drug-specific parameters of CLB and N-CLB used for the development of the final PBPK model.

Compound		CLB		N-CLB	
	Parameter (Units)	Value	Reference	Value	Reference
Physiochemical Properties	Molecular weight (g/mol)	300.74	ChEMBL	286.71	ADMET Predictor
	LogP	2.14	ALOGPS	2.45	
	Compound Type	Monoprotic base		Monoprotic base	
	pKa	6.65	ChEMBL	3.93	
	B/P ratio	0.69	ADMET Predictor	0.69	ADMET Predictor
	f_u,p_	0.1	[24]	0.11	[24]
Absorption	Absorption Model	First-Order Absorption	Simcyp	First Order Absorption	Simcyp
	f_a_	0.93	[38]		
	k_a_ (1/h)	2.11 (Fasted) 1.25 (Fed)	[19] Estimated to capture clinical data		
	f_u,gut_	0.1	Assumed as f_u,p_	0.11	Assumed as f_u,p_
Distribution	Distribution Model	Full PBPK	The Rodgers and Rowland method	Full PBPK	The Rodgers and Rowland method
	V_ss_ (L/kg)	0.56	Predicted	1.04	Predicted
	K_p_ scalar	1	No adjustment needed to capture observed V_ss_	0.8	Optimized to fit clinical data
Elimination	CL_h_ (L/h)	2	[3]	1.09	[28]
	CL_R_ (L/h)	0.05	[26]	0.08	[28]
	Elimination Model	Enzyme Kinetics		Enzyme Kinetics	
	CL_int,CYP2C19_ (µL/min/nmol P450)	0.173	Back-calculated using the well-stirred liver model	0.636	Back-calculated using the well-stirred liver model
	CL_int,CYP3A4_ (µL/min/nmol P450)	0.019	Back-calculated using the well-stirred liver model		
	CL_int,CYP2B6_ (µL/min/nmol P450)	0.022	Back-calculated using the well-stirred liver model		

Abbreviations: CL_h_, hepatic clearance; CL_R_, renal clearance; CLB: clobazam; pKa: acid dissociation constant; LogP: partition coefficient; B/P: blood to plasma ratio; V_ss_: volume of distribution at the steady state; K_p_: tissue to plasma partition coefficient; Cl_int_: intrinsic clearance.

**Table 2:** Drug-specific parameters of STP used for the development of the final PBPK model.

	Compound	STP	
	Parameter (Units)	Value	Reference
**Physiochemical properties**	Molecular weight (g/mol)	234.29	PubChem
	LogP	2.94	[15]
	Compound type	Neutral	ADMET predictor
	B/P ratio	0.58	[41]
	f_u,p_	0.01	
**Absorption**	Absorption model	First Order Absorption	Simcyp
	f_a_	0.82	[40]
	k_a_ (1/h)	1.4	Fitted to capture C_max_
	f_u,gut_	0.01	Assumed as f_u,p_
	Lag time (h)	0.5–1	Fitted to capture t_max_
**Distribution**	Distribution model	Full PBPK	The Rodgers and Rowland method
	V_ss_ (L/kg)	1.74	Predicted
	K_p_ scalar	4.2	Predicted
**Elimination**	CL_po_ (L/h)	8–70	Fitted ^a^
	f_m, CYP1A2_	0.2	[8]
	f_m, CYP2C19_	0.15	
	f_m, CYP2C9_	0.08	
	f_m, CYP2D6_	0.075	
	f_m, CYP3A4_	0.14	
	f_m, CYP3A5_	0.11	
**Interaction** ^b^	CYP1A2 k_i_ (μM)	3.3	[8]
	CYP2B6 k_i_ (μM)	7	[8]
	CYP2C19 k_i_ (μM)	0.0139	Optimized ^c^
	CYP2C8 k_i_ (μM)	3.4	[8]
	CYP2C9 k_i_ (μM)	65	[8]
	CYP2D6 k_i_ (μM)	9.3	[10]
	CYP3A4 k_i_ (μM)	2.5	[48]
	P-gp k_i_ (μM)	46	[8]
	BCRP k_i_ (μM)	1.17	[8]

Abbreviations: BCRP, Breast Cancer Resistance Protein; P-gp, P-glycoprotein; STP: stiripentol; LogP: partition coefficient; B/P: blood to plasma ratio; V_ss_: volume of distribution at the steady state; K_p_: tissue to plasma partition coefficient; CL_po_: oral clearance; P-gp: p-glycoprotein; BCRP: breast cancer resistance protein. ^a^ Due to STP non-linearity, lower clearance values were used at higher doses and following chronic administration [Table S3]. ^b^ In vitro k_i_ values were reported in the literature as total k_i_; therefore, f_inc_ values of 1 were assumed. ^c^ The in vitro value was reported as 0.139 μM [49].

**Table 3:** Simulated and observed drug–drug interaction studies between CLB and STP in Dravet syndrome pediatric populations.

Study	Role in PBPK Model	CYP2C19 Phenotype	Dosing Regimen	Compound	Parameter	Simulated	Observed	Simulated/Observed
**Chiron et al., 2000** [13]	DDI simulation between STP + CLB in pediatrics	Not reported	STP 25 mg/kg BID, CLB 1 mg/kg QD	CLB	C_min_,_inh_ (mg/L)	0.88	0.84 (0.66–1.02)	1.05
					C_min_ ratio	1.77	1.9	0.93
				N-CLB	C_min_,_inh_ (mg/L)	9.00	11.6 (10.3–12.9)	0.78
					C_min_ ratio	7.75	5.5	1.4
**Inoue et al., 2014** [45]	DDI simulation between STP + CLB in Japanese population	EMs	STP 25 mg/kg BID, CLB 1 mg/kg QD	CLB	C_min_,_inh_ (mg/L)	0.94	0.52 ± 0.28	1.81
					C_min_ ratio	1.64	1.86	0.88
				N-CLB	C_min_,_inh_ (mg/L)	10.03	7.50 ± 3.58	1.34
					C_min_ ratio	7.9	6.1	1.30
		PMs		CLB	C_min_,_inh_ (mg/L)	1.08	0.72 ± 0.57	1.5
					C_min_ ratio	1.31	1.71	0.77
				N-CLB	C_min_,_inh_ (mg/L)	13.29	10.07 ± 3.53	1.32
					C_min_ ratio	0.90	0.62	1.45
**Yamamoto et al., 2020** [46]		EMs	STP 17.5 mg/kg BID, CLB 1 mg/kg QD	CLB	C_min_,_inh_ (mg/L)	0.91	0.59 ± 0.07	1.54
					C_min_ ratio	1.54	1.79	0.86
				N-CLB	C_min_,_inh_ (mg/L)	8.91	6.38 ± 0.49	1.40
					C_min_ ratio	7.14	4.73	1.51
		PMs		CLB	C_min_,_inh_ (mg/L)	1.01	0.66 ± 0.08	1.53
					C_min_ ratio	1.22	1.27	0.96
				N-CLB	C_min_,_inh_ (mg/L)	12.75	10.26 ± 0.98	1.24
					C_min_ ratio	0.93	0.63	1.48

Observed data are presented as mean (95% CI) for Chiron et al. and as mean ± standard deviation for Inoue et al. and Yamamoto et al.; C_min,inh_ is the minimum plasma concentration observed in the presence of an inhibitor, whereas C_min ratio_ is the ratio of the minimum plasma concentration in the presence of the inhibitor to that in the absence of the inhibitor (C_min,inh_/C_min,control_); all C_min_ values for CLB and N-CLB are dose-normalized. Abbreviations: BID, twice daily; QD, once daily; DDI: drug-drug interaction; STP: stiripentol; EMs: extensive metabolizers; PMs: poor metabolizers; CLB: clobazam.

## Data Availability

The original contributions presented in this study are included in the article and Supplementary Material. Further inquiries can be directed to the corresponding author.

## Supplementary Materials

The following supporting information can be downloaded at: https://www.mdpi.com/article/10.3390/jpm15110549/s1, Table S1: Intrinsic clearance (Cl_int_) values estimated from the well-stirred liver model for clobazam (CLB) PBPK Model. Table S2: Summary of noncompartmental pharmacokinetic parameters for *n*-desmethylclobazam (N-CLB) following a 30 mg oral dose in healthy subjects. Table S3: Fitted oral clearance (CL_po_) values for STP across different dosing regimens. Table S4: Comparison of simulated and observed pharmacokinetic parameters for CLB across clinical studies. Table S5: Comparison of simulated and observed pharmacokinetic parameters for N-CLB across clinical studies. Table S6: Comparison of simulated and observed pharmacokinetic parameters for STP across clinical studies. Table S7: Comparison of predicted and observed pharmacokinetic parameters for CLB, N-CLB, and STP in a pediatric population. Figure S1: Goodness-of-fit plot of predicted versus observed pharmacokinetic metrics (AUC, C_max_, C_min_) for STP across various clinical studies. The solid and dash lines represent the identity line and 2-fold deviation, respectively. Figure S2: Predicted versus observed plasma concentration-time profiles of STP following multiple-dose administration in different pediatric age groups. Figure S3: Predicted versus observed minimum plasma concentrations (C_min_) of CLB and N-CLB in extensive (EM) and poor metabolizers (PM).
