# Supplementary material for: Physiologically Based Pharmacokinetic Modeling of Clobazam and Stiripentol Co-Therapy in Dravet Syndrome
Source: J Pers Med. 2025 Nov 11;15(11):549. doi: 10.3390/jpm15110549 (PMC12653854; doi:10.3390/jpm15110549)
Supplement: Supplementary file 1 [file jpm-15-00549-s001.zip › jpm-3931380-supplementary.pdf]

Supplementary Material

Table S1. Intrinsic clearance (Cl_{int}) values estimated from the well-stirred liver model for clobazam (CLB) PBPK Model.

Compound	Parameter	Value	Reference
**CLB**	CL_{po} (L/h)	2	[1]
	CL_{Add} (L/h)	0.6	Calculated (30% of CL_{po})
	% Hep met CL	70%	[2]
	CYP3A4 CL_{int} (μL/min/pmol)	0.019	Back calculated using well-stirred liver model
	% Hep met CL	19%	[2]
	CYP2C19 CL_{int} (μL/min/pmol)	0.173	Back calculated using well-stirred liver model
	% Hep met CL	11%	[2]
	CYP2B6 CL_{int} (μL/min/pmol)	0.022	Back calculated using well-stirred liver model
**NCLB**	CL_{po} (L/h)	1.09	[3]
	% Hep met CL	100%	Assumed
	CYP2C19 CL_{int} (μL/min/pmol)	0.636 for SD	Back calculated using well-stirred liver model

Cl_{int}, intrinsic clearance; CL_{po}, oral clearance; SD, single dose; MD, multiple dose; CLB: clobazam; NCLB: N-desmethylclobazam; CLAdd: additional clearance; .

Estimation of CLB k_a from Greenblatt 1981 Study [4]

Average ka reported in the study = 19.75 min

$$ka = \frac{0.693}{\frac{t1}{t}a} = \frac{0.693}{19.75\,\text{min}/60} = 2.11\ h^{-1}$$

Estimation of V_{ss} , CL_{po} and CL_{renal} for N-CLB from Pullar 1987 Study [3]

Plasma concentration-time profiles for N-CLB following a 30 mg oral dose in healthy subjects were digitized from Pullar et al. (1987) and analyzed using PKPlus® (Simulation Plus, Inc., Lancaster, CA).

Table S2. Summary of noncompartmental pharmacokinetic parameters for N-desmethylclobazam (N-CLB) following a 30 mg oral dose in healthy subjects.

Parameter	Symbol	Value	Units	Calculation / Source
**Elimination rate constant**	k_{el}	0.013	h^{-1}	PKPlus (terminal phase)
**Area Under the curve**	AUC_0–∞	27.57	μg·h/mL	PKPlus
**Area Under the first moment curve**	$AUMC_0$–∞	2197.9	μg·h²/mL	PKPlus
**Mean residence time**	MRT = AUMC/AUC	79.71	h	PKPlus calculation
**Apparent oral clearance**	CL/F = Dose/AUC	1.09	L/h	PKPlus output
**Apparent Vss/F**	(CL/F)×MRT	86.72	L	Derived from PKPlus parameters

Calculate CL_R.; Vss/F: apparent volume of distribution at steady state; AUC: area under the curve; MRT: mean residence time; AUMC: area under the moment curve; CL/F: apparent clearance;

Calculate CL_R

$$CLrenal = \text{CLpo} * \text{ fe} = 1.09 * 0.08 = 0.08 \text{ L/h}$$

Table S3. Fitted oral clearance (CL_{po}) values for stiripentol (STP) across different dosing regimens.

	**Dose**	**Study**	**CL_{po} (L/h)**
**SD**	300 mg	Levy et al., 1983 [5]	70
	500-600 mg	Levy et al., 1983 [5], Peigne et al., 2014 [6]	60
	1000-1200 mg	Levy et al., 1983 [5], Peigne et al., 2014 [6]	35
	2000 mg	Peigne et al., 2014 [6]	25
**MD**	1200-1800/day given as divided doses	Levy et al., 1983[a] [5], Levy et al., 1984[b] [7], Morrison et al., 2020[c] [8]	17
	1500 BID	Tran et al., 1997 [9]	8
**Pediatrics**	19 mg/kg BID[d]	May et al., 2012 [10]	14
	18 mg/kg BID[e]		10
	16 mg/kg BID[f]		8
	17.5-25 mg/kg BID	Chiron et al., 2000[g] [11], Inoue et al., 2014[h] [12], Yamamoto et al., 2020[i] [13]	8

[a]1200 mg/day given as four daily doses (200,300,300, and 400), [b]200 mg TID day 1-3, 400 mg TID day 3-7, and 600 mg TID day 7-14, [c]750 mg BID. [d]<6 years age group, [e]6-11.9 years age group, [f]12-25 years age group (older children and adolescents receive higher absolute STP doses, resulting in reduced clearance). [g]25 mg/kg BID, age range: 3-16.7 years, [h]25 mg/kg BID age range: 1-24 years, [i]17.5 mg/kg BID age range: 1-25 years. BID, twice daily; CL_{po}, oral clearance, MD, multiple dose; SD, single dose.

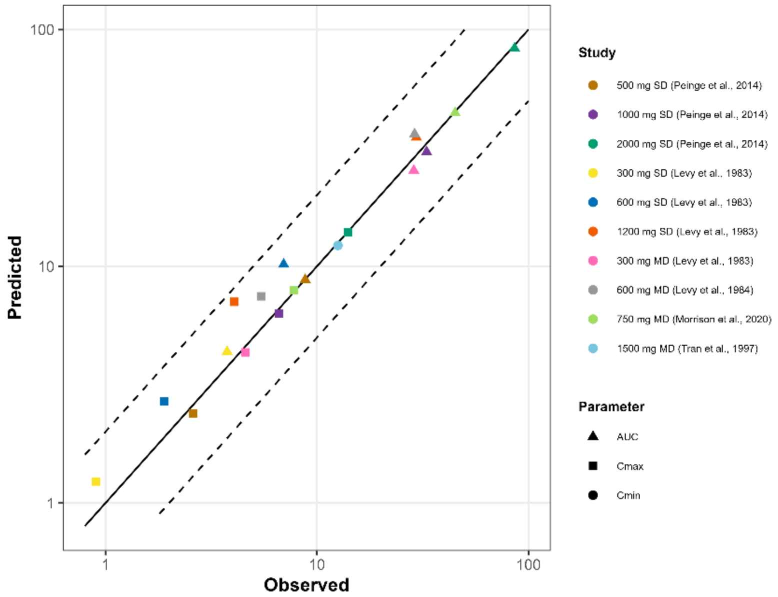

Figure S1. Goodness-of-fit plot of predicted versus observed PK metrics (AUC, C_{max}, C_{min}) for STP across various clinical studies [5-9]. The solid and dash lines represent the identity line and two-fold deviation, respectively. Abbreviations: Abbreviations: MD, multiple dose; SD, single dose.

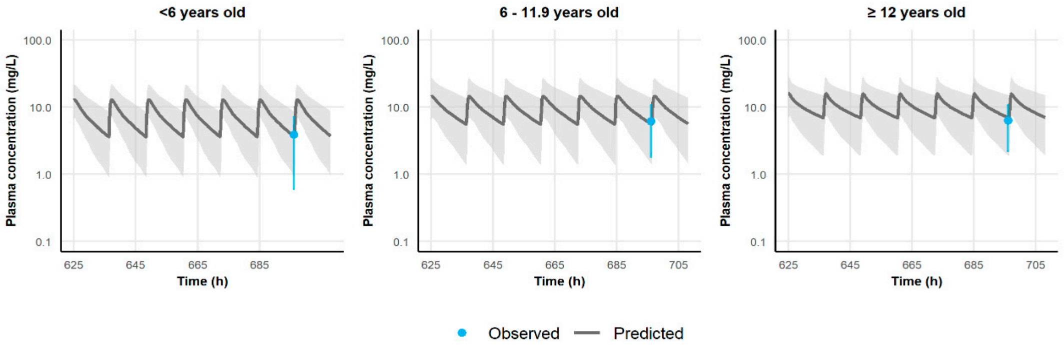

Figure S2. Predicted versus observed plasma concentration-time profiles of STP following multiple-dose administration in different pediatric age groups. The grey line represents the simulated mean concentration profile, and the grey-shaded area represents the simulated 5th to 95th percentile. Blue circles represent observed data from the study used in pediatric PBPK model development [10].

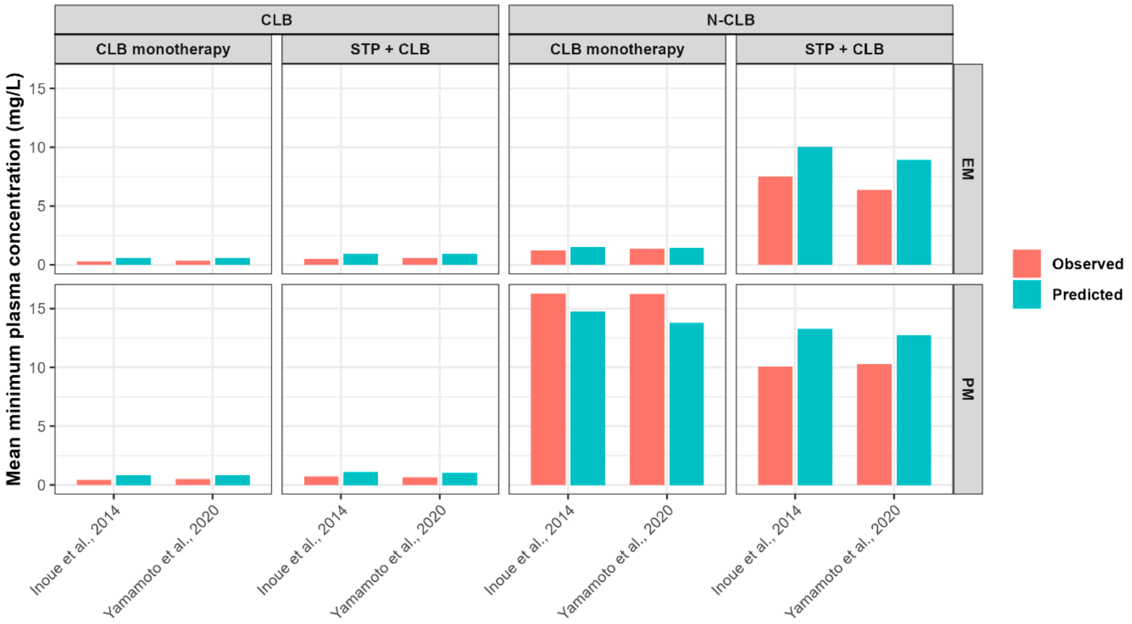

Figure S3. Predicted versus observed minimum plasma concentrations (C_{min}) of CLB and N-CLB in extensive (EM) and poor metabolizers (PM) [12,13].

Table S4. Comparison of simulated and observed pharmacokinetic parameters for CLB across clinical studies.

Study	Role in PBPK model	Number of subjects	Age (years)	% of females	Dosing Regimen	Parameter	Simulated	Observed	Simulated/Observed
**Bun et al. 1986 [14]**	Model Development	6	18-29	0	20 mg SD	AUC_{0-inf}	8946	6969	1.28
						C_{max}	458	351 ± 58	1.30
**Greenblatt et al. 1983 [15]**		12	19-37	42	20 mg SD	AUC_{0-inf}	9197	8549	1.08
						C_{max}	483	480*	1.01

**Divoll et al. 1982 [16]**		12	22-34	0	20 mg SD (Fasted)	AUC_{0-inf}	8911	10200	0.87
						C_{max}	459	465	0.99
		12	22-34	0	20 mg SD (Fed)	AUC_{0-inf}	8961	10400	0.86
						C_{max}	410	333	1.23
**Greenblatt et al. 1980 [17]**	Model Verification	1	30	0	20 mg SD	AUC_{0-inf}	8871	9372*	0.95
						C_{max}	460	510	0.90
**Greenblatt et al. 1981 [4]**	Model Verification	8	20-37	0	20 mg SD	AUC_{0-inf}	9350	7248	1.29
						C_{max}	478	408	1.17
**Monjanel et al. 1994 [18]**	Model Verification	6	24-29	50	20 mg SD	AUC_{0-inf}	8898	7143	1.25
						C_{max}	458	350	1.31
**Cenraud et al. 1983 [19]**	Model Verification	6	23-30	33	20 mg SD (Fasted)	AUC_{0-inf}	9131	11000 ± 4400	0.83
						C_{max}	478	491 ± 103	0.97
					20 mg SD (Fed)	AUC_{0-inf}	9180	10300 ± 4200	0.89
						C_{max}	421	362 ± 102	1.16
**Tolbert et al. 2019 [1]**	Model Verification	30	20-40	50	30 mg SD	AUC_{0-inf}	9200	10920	0.84
						C_{max}	472	313*	1.51
**Jawad et al. 1984 [20]**	Model Verification	6	18-31	17	30 mg SD	AUC_{0-inf}	13641	13741 ± 1867	0.99
						C_{max}	699	652	1.07
**Pullar et al. 1987 [3]**	Model Verification	8	23-40	0	30 mg SD	AUC_{0-inf}	13558	13000	1.04
						C_{max}	682	584 ± 49	1.17
**Walzer et al. 2012 [21]**	Model Verification	18	18-45	72	40 mg SD	AUC_{0-24}	12363	8783	1.41
						C_{max}	972	848	1.15
**Tedschi et al. 1983 [22]**	Model Verification	6	23-27	50	10 mg SD	AUC_{0-inf}	4667	4564 ± 859	1.02
						C_{max}	242	261 ± 24	0.93
**Vallner et al. 1980 [23]**	Model Verification	12	18-26	0	10 mg SD	AUC_{0-144}	4488	2162 ± 1588	2.08
						C_{max}	229	206 ± 72	1.11
					20 mg SD	AUC_{0-144}	8977	7065 ± 3180	1.27
						C_{max}	458	473 ± 197	0.97
					40 mg SD	AUC_{0-144}	17953	14356 ± 3674	1.25
						C_{max}	916	945 ± 348	0.97
**Bun et al. 1986 [14]**	Model Development	6	24-29	NA	10 mg BID for 28 days	C_{ssmin}	286	287 ± 63	1.00
**Greenblatt et al. 1983 [15]**	Model Development	7	20-37	0	10 mg oral dose once QD	C_{ssmin}	111	111 (55-216)	1.00

					for 22 days				
**Ochs et al. 1984 [24]**	Model Verification	13	19-59	53.8	5 mg BID for 22 days	C_{ssmin}	150	224	0.67
**Rupp et al. 1979 [25]**	Model Verification	10	26-51	0	10 mg BID for 28 days (Fed)	C_{ssmin}	286	512*	0.56
**Levy at al. 1983 [26]**	Model Verification	6	22-25	33.3	20 mg QD for 15 days	C_{ssmin}	210	354	0.59
**Walzer et al. 2012 [21]**	Model Verification	18	18-45	72	40 mg QD for 15 days	C_{max}	1401	1373	1.02
**Morrison et al. 2019 [b] [8]**	Model Verification	12	27.3 (10.9)	8.3	5 mg BID for 22 days	AUC_{0-t}	2399	2310	1.04
						C_{max}	250	239	1.05
**Walzer et al. 2012 [21]**	Model Verification	18	18-45	72	10 mg SD	AUC_{0-t}	4791	4360	1.10
						C_{max}	242	224	1.08
	DDI Simulation for CLB + Ketoconazole	17			400mg ketoconazole for 6 days, 10 mg CLB on day 6	$AUC_{0-inf\ inh}$	6471	6432	1.01
						$C_{max\ inh}$	253	190.1	1.33
						AUC ratio	1.39	1.53	0.91
						C_{max} Ratio	1.04	0.86	1.21
	Model Verification	18	22-41	33	10 mg SD	AUC_{0-inf}	4704	4239	1.11
						C_{max}	239	218	1.10
	DDI Simulation for CLB + omeprazole	18	22-41	33	40mg Omeprazole for 6 days, 10 mg CLB on day 6	$AUC_{0-inf\ inh}$	5030	5398	0.93
						$C_{max\ inh}$	240	211	1.14
						AUC ratio	1.07	1.3	0.82
						C_{max} Ratio	1.01	0.96	1.05

[a] Non-compartmental analysis was conducted to obtain the observed pharmacokinetic parameters. All values are arithmetic means ± SD, except for Morrison et al.,2019, which is presented as a geometric mean ± SD. All plasma concentrations are reported in ng/mL, and AUC values are in ng·h/mL. Abbreviations: AUC_{0-inf}, area under the concentration-time curve from time zero to infinity; AUC_t, area under the concentration-time curve over the dosing interval; BID, twice daily; QD, once daily; C_{max}, maximum plasma concentration; C_{ssmin}, minimum steady-state plasma concentration; C_{ss}, Mean steady-state plasma concentration; MD, multiple dose; SD, single dose; DDI: drug-drug interaction; CLB: clobazam.

Table S5. Comparison of simulated and observed pharmacokinetic parameters for N-CLB across clinical studies.

Study	Role in PBPK model	Number of subjects	Age (years)	% of females	Dosing Regimen	Parameter	Simulated	Observed	Simulated /Observed
**Bun et al. 1986 [14]**	Model Development	6	18-29	0	20 mg SD	$AUC_{0\text{-}inf}$	10536	10810*	0.97
						C_{max}	94	93 ± 21	1.01
**Greenblatt et al. 1983 [15]**		7	20-37	0	20 mg SD	$AUC_{0\text{-}72}$	7993	9337	0.86
						C_{max}	95	93*	1.02
**Divoll et al. 1982 [16]**		12	22-34	0	20 mg SD (Fasted)	$AUC_{0\text{-}t}$	9538	8800	1.08
						C_{max}	95	88	1.08
					20 mg SD (Fed)	$AUC_{0\text{-}t}$	9506	9500	1.00
						C_{max}	94	92	1.02
**Greenblatt et al. 1980**	Model Verification	1	30	0	20 mg SD	$AUC_{0\text{-}inf}$	9702	9799*	0.99
						C_{max}	95	91	1.04
**Greenblatt et al. 1981 [4]**	Model Verification	8	20-37	0	20 mg SD	$AUC_{0\text{-}t}$	5282	5261*	1.00
						C_{max}	91	73*	1.25
**Monjanel et al. 1994 [18]**	Model Verification	6	24-29	50	20 mg SD	$AUC_{0\text{-}t}$	8830	10700*	0.83
						C_{max}	94	96	0.98
**Tolbert et al. 2019 [1]**	Model Verification	30	20-40	50	30 mg SD	$AUC_{0\text{-}t}$	11129	13630	0.82
						C_{max}	87	75	1.16
**Jawad et al. 1984 [20]**	Model Verification	6	18-31	17	30 mg SD	$AUC_{0\text{-}t}$	27697	21227 ± 9244	1.30
						C_{max}	141	259	0.54
**Pullar et al. 1987 [3]**	Model Verification	8	23-40	0	30 mg SD	$AUC_{0\text{-}120}$	11668	13000	0.90
						C_{max}	137	126	1.09
**Walzer et al. 2012 [21]**	Model Verification	18	18-45	72	40 mg SD	$AUC_{0\text{-}t}$	2890	2363	1.22
						C_{max}	168	141	1.19
**Bun et al. 1986 [14]**	Model Development	6	24-29	NA	10 mg oral dose BID for 28 days	C_{ssmin}	580	1169 ± 588	0.50
**Greenblatt et al. 1983 [15]**	Model Development	7	20-37	0	10 mg oral dose once QD for 22 days	C_{ss}	277	164 (122-247)	1.69
**Ochs et al. 1984 [24]**	Model Verification	13	19-62	53.8	5 mg oral dose BID for 22 days	C_{ssmin}	284	513	0.55

**Rupp et al. 1979 [25]**	Model Verification	10	26-51	0	10 mg oral dose BID for 28 days (Fed)	C_{ssmin}	618	2751 [a]	0.22
**Levy at al. 1983 [26]**	Model Verification	6	22-25	33.3	20 mg QD for 15 days	C_{ssmin}	489	851	0.57
**Walzer et al. 2012 [21]**	Model Verification	18	18-45	72	40 mg oral dose QD for 15 days	C_{max}	1031	2566	0.40
**Morrison et al. 2019 [a] [8]**	Model Verification	12	27.3 (10.9)	8.3	5 mg BID for 22 days	AUC_{0-t}	3680	3720	0.90
						C_{max}	308	343	0.90
**Walzer et al. 2012 [21]**	Model Verification	18	19-45	72	10 mg SD	AUC_{0-inf}	5471	6987	0.78
						C_{max}	45	37.8	1.19
	DDI Simulation for CLB + Ketoconazole	18	19-45	72	400mg Ketoconazole for 6 days, 10 mg CLB on day 6	$AUC_{0-inf\ inh}$	4260	7851	0.54
						$C_{max\ inh}$	34	37.5	0.91
						AUC ratio	0.8	1.15	0.70
						C_{max} Ratio	0.77	0.99	0.78
	Model Verification	18	22-41	33	10 mg SD	AUC_{0-inf}	5483	5838	0.94
						C_{max}	46	39.2	1.17
	DDI Simulation for CLB + omeprazole	18	22-41	33	40mg Omeprazole for 6 days, 10 mg CLB on day 6	$AUC_{0-inf\ inh}$	6476	9176	0.71
						$C_{max\ inh}$	51	46.3	1.10
						AUC ratio	1.19	1.36	0.88
						C_{max} Ratio	1.1	1.15	0.96

[a] Non-compartmental analysis was conducted to obtain the observed pharmacokinetic parameters. All values are arithmetic means ± SD, except for Morrison et al.,2019, which is presented as a geometric mean ± SD. All plasma concentrations are reported in ng/mL, and AUC values are in ng·h/mL. Abbreviations: AUC_{0-inf}, area under the concentration-time curve from time zero to infinity; AUC_t, area under the concentration-time curve over the dosing interval; BID, twice daily; QD, once daily; C_{max}, maximum plasma concentration; C_{ssmin}, minimum steady-state plasma concentration; C_{ss}, Mean steady-state plasma concentration; MD, multiple dose; SD, single dose; DDI: drug-drug interaction; CLB: clobazam.

Table S6. Comparison of simulated and observed pharmacokinetic parameters for STP across clinical studies.

Study	Role in PBPK model	Number of subjects	Age (years)	% of females	Food status	Dosing regimen	Parameter	Simulated	Observed	Simulated/Observed
		12	18-40	0	Fed	500 mg	AUC_{0-inf}	8.79	8.8 ± 3.7	1.00

**Peinge et al., 2014 [6]**	SD model development						C_{max}	2.39	2.6 ± 1.2	0.92
		12	18-40	0		1000 mg	AUC_{0-inf}	30.42	33 ± 11	0.92
							C_{max}	6.32	6.6 ± 1.8	0.96
		12	18-40	0		2000 mg	AUC_{0-inf}	83.28	86 ± 27	0.97
							C_{max}	13.94	14 ± 4.8	1.00
**Levy et al., 1983[a] [5]**	SD model verification	6	29-43	0	Fasted	300 mg	AUC_{0-inf}	4.36	3.76	1.16
							C_{max}	1.23	0.9	1.37
		6	29-43	0		600 mg	AUC_{0-inf}	10.19	6.96	1.46
							C_{max}	2.69	1.9	1.42
		6	29-43	0		1200 mg	AUC_{0-inf}	35.03	29.53	1.19
							C_{max}	7.08	4.07	1.74
**Morrison et al., 2019 [8]**	MD model development	12	35.1 (±12.6)	0.42	Fed	750 mg BID for 14 days	AUC_t	44.48	45 ±115	0.99
							C_{max}	7.94	7.8 ±2	1.02
**Levy et al., 1983[a] [5]**	MD model verification	6	30-43	0	Fasted	1200mg/day divided into four doses for 8 days	AUC_t	25.39	28.69	0.88
							C_{max}	4.33	4.58	0.95
**Levy et al., 1984[b] [7]**	MD model verification	6	25-34	0	Fed[d]	200 mg TID day 1-3, 400 mg TID day 3-7, and 600 mg TID day 7-14	AUC_t	36.21	28.95[c]	1.25
							C_{max}	7.47	5.44[c]	1.37
**Tran et al., 1997 [9]**	MD model verification	12	21-29	0.5[d]	Fed[d]	500 mg BID day 1, 1000 mg BID day 2, 1500 mg BID day 3-14	C_{min}	12.25	12.54 ± 3.91	0.98

[a] Non-compartmental analysis was conducted to obtain the observed pharmacokinetic parameters. [b] Results are shown for the third dosing interval (600 mg TID, days 7–14). [c] Mean of two subjects. [d] Assumed. Simulated values represent arithmetic means, while observed values are arithmetic means ± SD, except for Morrison et al.,2019, which is presented as a geometric mean ± SD. C_{max} and C_{min} are reported in mg/L, and AUC values are in mg·h/L. Abbreviations: AUC_{0-inf}, area under the concentration-time curve from time zero to infinity; AUC_t, area under the concentration-time curve over the dosing interval; BID, twice daily; C_{max}, maximum plasma concentration; C_{min}, minimum plasma concentration; MD, multiple dose; SD, single dose; TID, three times daily.

Table S7. Comparison of predicted and observed pharmacokinetic parameters for CLB, N-CLB, and STP in pediatric population.

Study	Role in PBPK model	CYP2C19 phenotype	N	Age (years)	% of females	Dosing regimen	Compound	Parameter	Simulated	Observed	Simulated/Observed
**Chiron et al., 2000 [11]**	CLB model development in pediatrics	Not reported	21	3-16.7	0.7	CLB 1 mg/kg QD	CLB	C_{min}	0.55	0.45 (0.39-0.51)	1.22
							N-CLB	C_{min}	1.65	2.1 (1.3-2.9)	0.79
**Inoue et al., 2014 [12]**	CLB model verification in pediatrics Japanese population	EM	17	1-24	0.375	CLB 1 mg/kg QD	CLB	C_{min}	0.58	0.29 ± 0.14	2
							N-CLB	C_{min}	1.52	1.23 ± 0.66	1.24
		PM	3				CLB	C_{min}	0.83	0.42 ± 0.18	1.98
							N-CLB	C_{min}	14.75	16.28 ± 5.00	0.91
**Yamamoto et al., 2020 [13]**	CLB model verification in pediatrics Japanese population	EM	28	1-25[a]	0.67	CLB 1 mg/kg QD	CLB	C_{min}	0.60	0.33 ± 0.03	1.82
							N-CLB	C_{min}	1.47	1.35 ± 0.17	1.09
		PM	10				CLB	C_{min}	0.83	0.52 ± 0.11	1.6
							N-CLB	C_{min}	13.77	16.23 ± 1.31	0.85
**May et al., 2012 [10]**	STP model development in pediatrics	Not reported	20	<6	0.45	19 mg/kg BID	STP	C_{min}	3.53	3.9 ±3.3	0.91
			16	6-11.9	0.31	18 mg/kg BID	STP	C_{min}	5.48	6.2 ±4.4	0.88
			39	≥ 12[b]	0.56	16 mg/kg BID	STP	C_{min}	7.98	6.4 ±4.2	1.25
**Chiron et al., 2000 [11]**	STP model verification in pediatrics	Not reported	21	3-16.7	0.7	25 mg/kg BID	STP	C_{min}	10.6	10 (8.3,11.7	1.06

[a]Age range in study 1: 1–40 years, [b]age range in study: >12–45 years; however, all simulations were capped at 25 years since 25 is the maximum age in the Simcyp pediatric populations. All simulated and observed C_{min} values were dose-normalized for CLB and N-CLB and are reported in mg/L. Simulated values represent arithmetic means, while observed values are reported as follows: Chiron et al. as mean (95% CI), May et al. and Inoue et al. as mean (±SD), and Yamamoto et al. as mean (±SE). Abbreviations: BID, twice daily; C_{min}, minimum plasma concentration; EM, extensive metabolizer; PM, poor metabolizer; QD, once daily; CLB: clobazam; STP: stiripentol.

References

1. Tolbert, D.; Larsen, F. A comprehensive overview of the clinical pharmacokinetics of clobazam. *The Journal of Clinical Pharmacology* **2019**, *59*, 7-19.
2. Giraud, C.; Tran, A.; Rey, E.; Vincent, J.; Tréluyer, J.-M.; Pons, G. IN VITRO CHARACTERIZATION OF CLOBAZAM METABOLISM BY RECOMBINANT CYTOCHROME P450 ENZYMES: IMPORTANCE OF CYP2C19. *Drug Metabolism and Disposition* **2004**, *32*, 1279-1286, doi:10.1124/dmd.32.11.1279.
3. Pullar, T.; Haigh, J.R.; Peaker, S.; Feely, M.P. Pharmacokinetics of N-desmethylclobazam in healthy volunteers and patients with epilepsy. *Br J Clin Pharmacol* **1987**, *24*, 793-797, doi:10.1111/j.1365-2125.1987.tb03247.x.
4. Greenblatt, D.J.; Divoll, M.; Puri, S.K.; Ho, I.; Zinny, M.A.; Shader, R.I. Clobazam kinetics in the elderly. *British journal of clinical pharmacology* **1981**, *12*, 631-636, doi:10.1111/j.1365-2125.1981.tb01281.x.
5. Levy, R.H.; Lin, H.S.; Blehaut, H.M.; Tor, J.A. Pharmacokinetics of stiripentol in normal man: evidence of nonlinearity. *J Clin Pharmacol* **1983**, *23*, 523-533, doi:10.1002/j.1552-4604.1983.tb01799.x.
6. Peigné, S.; Rey, E.; Le Guern, M.E.; Dulac, O.; Chiron, C.; Pons, G.; Jullien, V. Reassessment of stiripentol pharmacokinetics in healthy adult volunteers. *Epilepsy Res* **2014**, *108*, 909-916, doi:10.1016/j.eplepsyres.2014.03.009.
7. Levy, R.H.; Loiseau, P.; Guyot, M.; Blehaut, H.M.; Tor, J.; Moreland, T.A. Stiripentol kinetics in epilepsy: nonlinearity and interactions. *Clin Pharmacol Ther* **1984**, *36*, 661-669, doi:10.1038/clpt.1984.237.
8. Morrison, G.; Crockett, J.; Blakey, G.; Sommerville, K. A Phase 1, Open-Label, Pharmacokinetic Trial to Investigate Possible Drug-Drug Interactions Between Clobazam, Stiripentol, or Valproate and Cannabidiol in Healthy Subjects. *Clin Pharmacol Drug Dev* **2019**, *8*, 1009-1031, doi:10.1002/cpdd.665.
9. Tran, A.; Rey, E.; Pons, G.; Rousseau, M.; d'Athis, P.; Olive, G.; Mather, G.G.; Bishop, F.E.; Wurden, C.J.; Labroo, R.; et al. Influence of stiripentol on cytochrome P450-mediated metabolic pathways in humans: in vitro and in vivo comparison and calculation of in vivo inhibition constants. *Clin Pharmacol Ther* **1997**, *62*, 490-504, doi:10.1016/s0009-9236(97)90044-8.
10. May, T.W.; Boor, R.; Mayer, T.; Jürgens, U.; Rambeck, B.; Holert, N.; Korn-Merker, E.; Brandt, C. Concentrations of Stiripentol in Children and Adults With Epilepsy: The Influence of Dose, Age, and Comedication. *Therapeutic Drug Monitoring* **2012**, *34*, 390-397, doi:10.1097/FTD.0b013e31825dc4a6.
11. Chiron, C.; Marchand, M.C.; Tran, A.; Rey, E.; d'Athis, P.; Vincent, J.; Dulac, O.; Pons, G. Stiripentol in severe myoclonic epilepsy in infancy: a randomised placebo-controlled syndrome-dedicated trial. STICLO study group. *Lancet* **2000**, *356*, 1638-1642, doi:10.1016/s0140-6736(00)03157-3.
12. Inoue, Y.; Ohtsuka, Y. Effectiveness of add-on stiripentol to clobazam and valproate in Japanese patients with Dravet syndrome: additional supportive evidence. *Epilepsy Res* **2014**, *108*, 725-731, doi:10.1016/j.eplepsyres.2014.02.008.
13. Yamamoto, Y.; Takahashi, Y.; Ikeda, H.; Imai, K.; Kagawa, Y.; Inoue, Y. Impact of CYP2C19 Phenotypes on Clinical Efficacy of Stiripentol in Japanese Patients With Dravet Syndrome. *Therapeutic Drug Monitoring* **2020**, *42*, 302-308, doi:10.1097/ftd.0000000000000676.
14. Bun, H.; Coassolo, P.; Gouezo, F.; Serradimigni, A.; Cano, J.P. Time-dependence of clobazam and N-demethylclobazam kinetics in healthy volunteers. *Int J Clin Pharmacol Ther Toxicol* **1986**, *24*, 287-293.
15. Greenblatt, D.J.; Divoll, M.; Puri, S.K.; Ho, I.; Zinny, M.A.; Shader, R.I. Reduced single-dose clearance of clobazam in elderly men predicts increased multiple-dose accumulation. *Clin Pharmacokinet* **1983**, *8*, 83-94, doi:10.2165/00003088-198308010-00005.
16. Divoll, M.; Greenblatt, D.J.; Ciraulo, D.A.; Puri, S.K.; Ho, I.; Shader, R.I. Clobazam kinetics: intrasubject variability and effect of food on adsorption. *J Clin Pharmacol* **1982**, *22*, 69-73, doi:10.1002/j.1552-4604.1982.tb05711.x.
17. Greenblatt, D.J. Electron-capture GLC determination of clobazam and desmethylclobazam in plasma. *J Pharm Sci* **1980**, *69*, 1351-1352, doi:10.1002/jps.2600691131.
18. Monjanel-Mouterde, S.; Antoni, M.; Bun, H.; Botta-Frindlund, D.; Gauthier, A.; Durand, A.; Cano, J.P. Pharmacokinetics of a single oral dose of clobazam in patients with liver disease. *Pharmacol Toxicol* **1994**, *74*, 345-350, doi:10.1111/j.1600-0773.1994.tb01371.x.
19. Cenraud, B.; Guyot, M.; Levy, R.H.; Brachet-Liermain, A.; Morselli, P.L.; Moreland, T.A.; Loiseau, P. No effect of food intake on clobazam absorption. *Br J Clin Pharmacol* **1983**, *16*, 728-730, doi:10.1111/j.1365-2125.1983.tb02251.x.
20. Jawad, S.; Richens, A.; Oxley, J. Single dose pharmacokinetic study of clobazam in normal volunteers and epileptic patients. *Br J Clin Pharmacol* **1984**, *18*, 873-877, doi:10.1111/j.1365-2125.1984.tb02558.x.

21. Walzer, M.; Bekersky, I.; Blum, R.A.; Tolbert, D. Pharmacokinetic drug interactions between clobazam and drugs metabolized by cytochrome P450 isoenzymes. *Pharmacotherapy* **2012**, *32*, 340-353, doi:10.1002/j.1875-9114.2012.01028.x.
22. Tedeschi, G.; Riva, R.; Baruzzi, A. Clobazam plasma concentrations: pharmacokinetic study in healthy volunteers and data in epileptic patients. *Br J Clin Pharmacol* **1981**, *11*, 619-622, doi:10.1111/j.1365-2125.1981.tb01180.x.
23. Vallner, J.J.; Kotzan, J.A.; Stewart, J.T.; Honigberg, I.L.; Needham, T.E.; Brown, W.J. Plasma levels of clobazam after 10-, 20-, and 40-mg tablet doses in healthy subjects. *J Clin Pharmacol* **1980**, *20*, 444-451, doi:10.1002/j.1552-4604.1980.tb01717.x.
24. Ochs, H.R.; Greenblatt, D.J.; Lüttkenhorst, M.; Verburg-Ochs, B. Single and multiple dose kinetics of clobazam, and clinical effects during multiple dosage. *Eur J Clin Pharmacol* **1984**, *26*, 499-503, doi:10.1007/bf00542148.
25. Rupp, W.; Badian, M.; Christ, O.; Hajdú, P.; Kulkarni, R.D.; Taeuber, K.; Uihlein, M.; Bender, R.; Vanderbeke, O. Pharmacokinetics of single and multiple doses of clobazam in humans. *Br J Clin Pharmacol* **1979**, *7 Suppl 1*, 51s-57s, doi:10.1111/j.1365-2125.1979.tb04665.x.
26. Levy, R.H.; Lane, E.A.; Guyot, M.; Brachet-Liermain, A.; Cenraud, B.; Loiseau, P. Analysis of parent drug-metabolite relationship in the presence of an inducer. Application to the carbamazepine-clobazam interaction in normal man. *Drug Metab Dispos* **1983**, *11*, 286-292.
